# Silicon and potassium synergistically alleviate salt stress and enhance soil fertility, nutrition, and physiology of passion fruit seedlings

**DOI:** 10.3389/fpls.2025.1685221

**Published:** 2025-10-21

**Authors:** Alicia Camila Zeferino da Silva, Rennan Fernandes Pereira, Raquel da Silva Ferreira, Samuel Barbosa Alves, Franklin Suassuna de Sousa, Samuel Saldanha Rodrigues, José Felix de Brito Neto, Alberto Soares de Melo, Roseano Medeiros da Silva, Evandro Franklin de Mesquita

**Affiliations:** ^1^ Graduate Program in Agricultural Sciences, Paraíba State University, Campina Grande, Brazil; ^2^ Department of Agrarian and Exact, Paraíba State University, Catolé do Rocha, Brazil; ^3^ Department of Biology, Paraíba State University, Campina Grande, Brazil

**Keywords:** *Passiflora edulis*, abiotic stress, water salinity, silicic acid, potassium sulfate

## Abstract

**Introduction:**

Yellow passion fruit (*Passiflora edulis*) is widely cultivated in Brazil but suffers adverse effects when irrigated with saline water, a common condition in the Brazilian semiarid region. Silicon and potassium have been extensively studied as salt stress mitigators, yet little is known about the synergistic effects between these two elements. Therefore, we evaluated the synergistic effects of silicon and potassium on alleviating salt stress in yellow passion fruit seedlings.

**Methods:**

The experiment was conducted in a greenhouse using a completely randomized 4 × 2 + 2 factorial design with five replicates. Four doses of silicic acid (1.26, 2.52, 3.78, and 5.04 g dm^-3^) and two potassium doses (150 and 600 mg dm^-3^) were tested, with two controls (saline and non-saline water). Soil fertility (pH, electrical conductivity, mineral elements) and the following plant variables were assessed: foliar concentrations of macro- and micronutrients, biochemical traits (chlorophyll and proline), gas exchange, relative water content, electrolyte leakage, growth, and biomass accumulation. ANOVA (F-test) was performed, with regression and Dunnett’s test used for significant effects.

**Results:**

The silicon-potassium combination reduced soil pH and electrical conductivity, while increasing the availability of P, K, Ca, Mg, S, Fe, Mn, Zn, and Cu. Foliar nutrient concentrations improved while leaf Na^+^ decreased. Biochemically, there was a significant increase in total chlorophyll, along with reduced proline levels. Plants also exhibited higher CO_2_ assimilation, stomatal conductance, and relative water content, with reduced electrolyte leakage. Plant height and shoot and root dry masses increased in response to silicon doses, with gains of up to 133% compared to the saline control.

**Discussion:**

Silicon and potassium acted synergistically to reduce soil and leaf salinity, improve nutrient availability, and enhance plant biochemical and physiological performance, leading to greater growth and biomass accumulation. The results support the combined application of silicon and potassium as an effective strategy to mitigate salt stress and promote the nutrition, physiology, and growth of yellow passion fruit seedlings under saline irrigation.

## 1 Introduction

*Passiflora edulis* f. *flavicarpa* Deg., commonly known as yellow passion fruit, is the most widely cultivated *Passiflora* species in Brazil. This species is valued for its high yield potential and favorable physicochemical fruit characteristics, which are largely enhanced by the country’s predominant edaphoclimatic conditions (Santos et al., 2021; Souto et al., 2022; Porto et al., 2025). However, the success of its cultivation depends directly on these environmental conditions and on appropriate management practices, starting from the seedling stage. In semiarid regions, characterized by high temperatures, low humidity, and water scarcity, the use of irrigation water with high electrical conductivity can hinder seedling establishment, adversely affecting water uptake, ionic balance, mineral nutrition, and, consequently, plant growth and productivity (Lima et al., 2023; Almeida et al., 2024).

Salinity is considered one of the main abiotic stresses affecting irrigated agriculture, as it induces osmotic and nutritional imbalances and causes damage to cell membrane integrity and the functioning of the photosynthetic apparatus (Liu et al., 2019; Ondrasek et al., 2022). In semiarid regions, the limited availability of freshwater and the recurrent use of saline water further exacerbate the detrimental effects of salinity on soils and plants (Diniz et al., 2021; Pessoa et al., 2022).

In this context, the use of stress-attenuating elements such as silicon (Si) has been increasingly investigated. Although not classified as essential, Si is recognized as a beneficial element for many plant species. It contributes to the activation of morphophysiological and biochemical mechanisms associated with stress tolerance, including cell wall reinforcement, increased antioxidant activity, stomatal regulation, improved water-use efficiency, and enhanced nutrient uptake (Ahmed et al., 2023; Queiroz et al., 2025).

In fruit crops, silicon has been recognized as an important element for mitigating the detrimental effects of abiotic stress. In mango, El-Dengawy et al. (2021) reported that irrigation with saline water combined with foliar nano-silicon increased leaf pigments, soluble carbohydrates, total phenols, and essential nutrients such as Mg, N, P, and K, while simultaneously reducing the severity of salinity-induced damage. In strawberry, Yaghubi et al. (2019) found that potassium silicate restored dry mass distribution in salt-stressed plants, decreased Na uptake in leaves, and increased total soluble solids and titratable acidity in the fruits. In cantaloupe, Alam et al. (2021) observed that silicic acid application under water stress enhanced pulp thickness and soluble solids content. In yellow passion fruit, Si application has been linked to increased epidermal thickness, improved photosynthetic performance, and greater biomass accumulation even under salt stress (Costa et al., 2016; Diniz et al., 2021; Almeida et al., 2024). Futhermore, Si can modify the rhizospheric environment by complexing toxic ions and enhancing the availability of soluble nutrient in the soil (Coskun et al., 2016; Dhiman et al., 2021).

Potassium (K^+^), in turn, is an essential macronutrient involved in osmotic regulation, stomatal function, enzymatic activation, and assimilate translocation (Taiz et al., 2017). In saline environments, K^+^ acts antagonistically to sodium (Na^+^), contributing to ionic balance and the maintenance of cellular metabolism (Wakeel, 2013; Souza et al., 2023). Applications of potassium silicate have shown potential to enhance Si and K^+^ uptake while limiting Na^+^ accumulation in plants, thereby increasing salt stress tolerance (Oraee and Tehranifar, 2023).

Recent studies have shown promising effects of combined or separate applications of silicon and potassium in fruit crops and other species under salinity. For example, Abidi et al. (2023) observed that pre-harvest foliar sprays of potassium-silicon in peach and nectarine improved fruit quality attributes such as firmness, soluble solids, phenolics, and anthocyanins, indicating that K–Si can enhance antioxidant mechanisms and physical properties related to oxidative stress. Similarly, in okra, studies have shown that separate or combined applications of silicon and potassium under saline irrigation increase dry matter, nitrogen uptake, and pod yield, demonstrating a synergistic effect of these elements in mitigating the impacts of salinity (Kurdali et al., 2022). Additionally, Alharby et al. (2022) reported that combined application of K and Si significantly increased Cd and Pb tolerance in quinoa, improving growth, stomatal conductance, and reducing oxidative stress, suggesting that the Si–K interaction could be exploited in tropical and subtropical fruit crops to mitigate the effects of salinity.

Despite these advances, studies investigating the combined effects of silicon and potassium on the physiological, nutritional, and biochemical performance of yellow passion fruit under saline irrigation remain limited. Moreover, little is also known about how this interaction affects soil fertility dynamics, which hinders the formulation of technical recommendations for saline environments. Therefore, the extrapolation of these findings to practical management strategies for passion fruit requires integrated studies. It is necessary to simultaneously evaluate plant physiological responses and soil chemical changes to support management practices in tropical and subtropical regions affected by salinity.

From this perspective, this study aimed to evaluate the synergistic effect of silicon and potassium application in mitigating salt stress in yellow passion fruit seedlings, with emphasis on physiological, biochemical, and nutritional responses, as well as on soil chemical attributes.

## 2 Materials and methods

### 2.1 Location and experimental conditions

The experiment was conducted from June to September 2024 in a greenhouse at the Center for Human and Agricultural Sciences of the Paraíba State University, located in Catolé do Rocha, PB, Brazil. The plant material used was the yellow passion fruit cultivar ‘BRS Gigante Amarelo’, propagated from seeds. Initially, seeds were sown in polyethylene trays containing cells with a volume of 0.0125 cm^3^. Subsequently, the most vigorous seedlings with one pair of definitive leaves were selected and transplanted into polyethylene bags containing 3 dm^3^ of a 1:1 (v/v) mixture of soil and cattle manure.

The soil used was classified as an Entisol (Fluvent), according to USDA – United States Department of Agriculture (2014), with a sandy clay loam texture and the following physical characteristics: 831.5, 100.0, and 68.5 g kg^-1^ of sand, silt, and clay, respectively; bulk density = 1.53 g cm^-3^; particle density = 2.61 g cm^-3^; total porosity = 0.42 m^3^ m^-3^; flocculation degree = 1,000 kg dm^-3^; and moisture content at -0.01, -0.03, and -1.50 MPa matric potentials of 65, 49, and 28 g kg^-1^, respectively. Regarding fertility, the soil exhibited the following attributes: pH = 6.0; P = 16.63 mg dm^-3^; K^+^, Ca²^+^, Mg^2+^, and Na^+^ contents of 0.08, 1.09, 1.12, and 0.05 cmolc dm^-3^, respectively; sum of exchangeable bases = 2.34 cmolc dm^-3^; H^+^ + Al^3+^ = 1.24 cmolc dm^-3^; Al^3+^ = 0 cmolc dm^-3^; cation exchange capacity (CEC) = 3.58 cmolc dm^-3^; base saturation (V) = 65.36%; and organic matter = 13.58 g kg^-1^.

The cattle manure presented the following characteristics: pH (H_2_O) = 7.7; electrical conductivity = 6.09 dS m^-1^; organic matter = 36.2 dag kg^-1^; organic carbon = 166.9 g kg^-1^; N = 13.9 g kg^-1^; C/N ratio = 12; and contents of P, K^+^, Ca^2+^, Mg^2+^, and S of 3.2, 18.7, 16.2, 6.1, and 2.5 g kg^-1^, respectively. The manure’s CEC was 133.9 mmol dm^-3^, and micronutrient contents were as follows: B = 14.8 mg kg^-1^; Fe = 11,129.9 mg kg^-1^; Cu = 19.3 mg kg^-1^; Mn = 491.4 mg kg^-1^; and Zn = 65.3 mg kg^-1^. Silicon and Na^+^ levels were 12.5 and 3.5 g kg^-1^, respectively.

**Figure 1:**
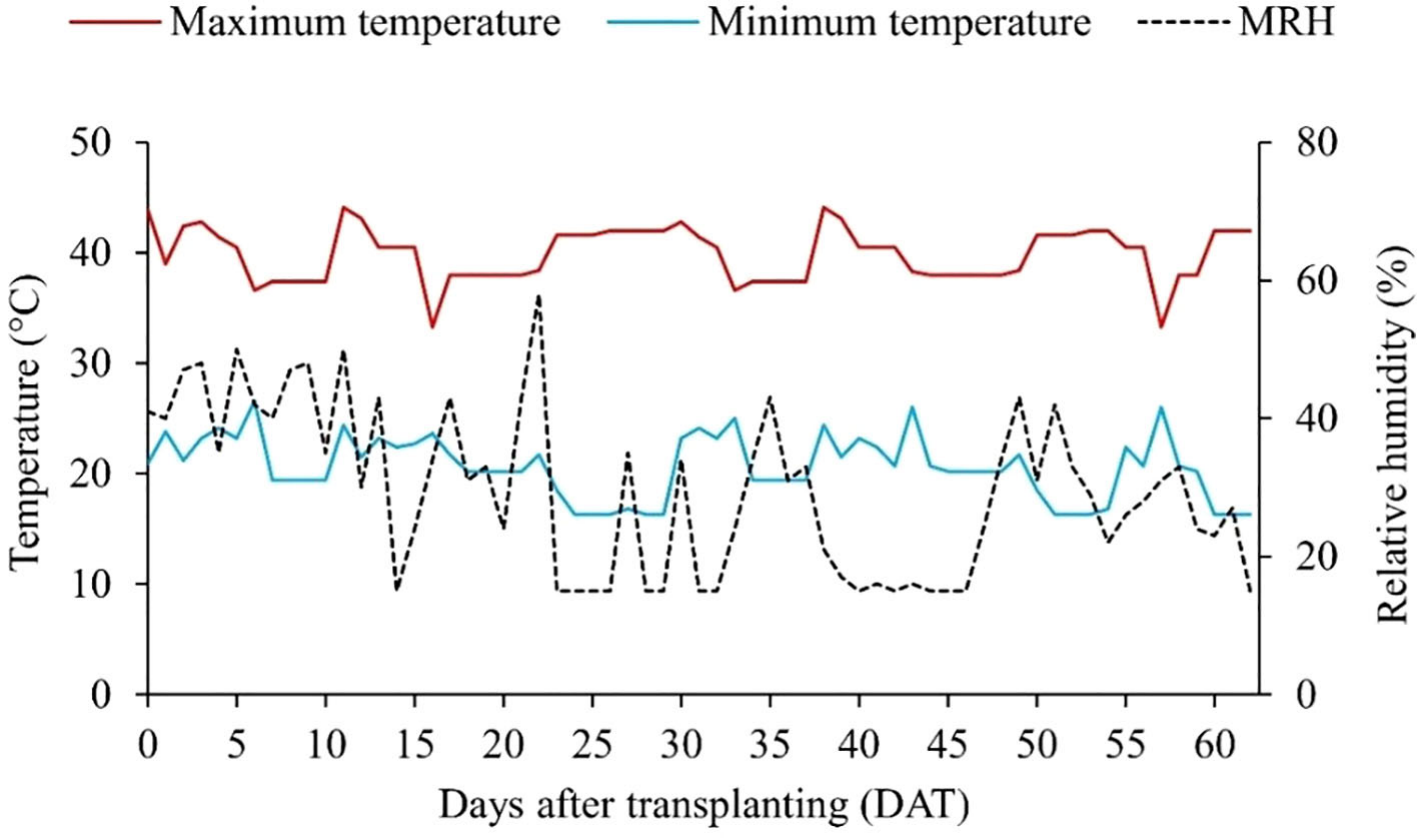
Maximum and minimum temperature, and mean relative humidity (MRH) recorded in the greenhouse during the experimental period.

The maximum and minimum temperatures, as well as the mean relative humidity recorded during the experimental period, are shown in **Figure 1**.

### 2.2 Treatments and experimental design

Five concentrations of silicic acid (1.26, 2.52, 3.78, and 5.04 g dm^-3^) were evaluated in combination with two potassium doses (150 and 600 mg dm^-3^). Additionally, two control treatments were included: one irrigated with saline water (Control 1) and the other with non-saline water (Control 2), both without chemical fertilization. The experimental design was completely randomized, arranged in a 4 × 2 + 2 factorial scheme, with five replications.

Plants receiving silicon and potassium treatments, as well as those in Control 1, were irrigated daily with water at an electrical conductivity (EC) of 4.0 dS m^-1^. Control 2 plants were irrigated with water at 0.5 dS m^-1^. The irrigation volume per plant per event was calculated based on the difference between the average container weight at maximum water-holding capacity and the average weight at the time of irrigation, divided by the total number of containers.

Elements were applied via fertigation, following the recommendations of Costa et al. (2016) and Almeida et al. (2006). Silicon was supplied using the commercial product Sifol^®^, composed of 92% SiO_2_ and 42.9% Si, with a bulk density ranging from 40 to 80 g L^-1^, particle size between 8 and 12 mesh, and pH ranging from 6.0 to 7.5. Potassium was provided in the form of potassium sulfate, containing 52% K_2_O and 18% S. Separate sources of silicon and potassium were used to isolate the independent contributions of these elements.

Silicon and potassium applications were divided into four stages: at base fertilization and at 15, 30, and 45 days after transplanting (DAT), with 25% of the total dose applied at each stage. To standardize sulfur input across treatments, a calibration was performed to ensure all plots received 0.57 g of sulfur per plant. In treatments with 150 mg dm^-3^ of potassium, this amount was added separately, whereas in treatments with 600 mg dm^-3^ of potassium, no additional sulfur was needed due to the concentration already present in the fertilizer.

### 2.3 Experimental analysis

#### 2.3.1 Soil fertility

At the end of the experiment, the substrate in which the plants were grown was analyzed for pH, electrical conductivity, macronutrient contents (P, K, Ca, Mg, and S), micronutrients (Fe, Cu, Mn, and Zn), and sodium (Na). The analyses followed the procedures described in the *Manual of Chemical Analysis of Soils, Plants and Fertilizers* (In Portuguese) by Embrapa– Empresa Brasileira de Pesquisa Agropecuária (2009).

#### 2.3.2 Leaf mineral element content

Sixty days after transplanting (DAT), eight leaves per plant were collected. The samples were washed with distilled water, oven-dried at 65 °C with forced air circulation until reaching constant weight, ground in a Wiley-type stainless steel knife mill, and stored in properly labeled, airtight containers.

Subsequently, the contents of macronutrients (N, P, K, Ca, Mg, and S), micronutrients (Fe, Zn, Cu, and Mn), sodium (Na), and silicon (Si) were determined following the methodologies compiled by Silva and Silva (2009). Nitrogen was quantified by the Kjeldahl method (dry digestion); phosphorus by spectrophotometry using the molybdenum blue complex; and silicon by a molybdenum blue spectrophotometric method adapted for this element.

#### 2.3.3 Biochemical analyses

Total chlorophyll content was quantified in the third leaf from the apex. Five leaf discs were macerated with 0.2 g of calcium carbonate and 5 mL of 80% acetone. The extract was centrifuged at 3,000 rpm for 10 minutes at 10°C, and the supernatant was transferred to a graduated cylinder and brought to a final volume of 5 mL using the same extracting solution. An aliquot was then placed in a cuvette for spectrophotometric reading at 646, and 663 nm. Total chlorophyll concentration was calculated using the equations proposed by Lichtenthaler (1987). All procedures were performed under controlled light conditions in a dark environment to prevent degradation of light-sensitive pigments.

Proline content was determined using 0.02 g of dry leaf matter placed in test tubes with 10 mL of distilled water. Samples were heated in a water bath at 100°C for 1 hour for extraction. Then, 1 mL of the extract was transferred to Falcon tubes, to which 1 mL of acid ninhydrin reagent and 1 mL of glacial acetic acid were added. Samples were vortexed for 20 seconds, sealed, and reheated in a water bath at 100°C for 1 hour. After this period, the reaction was stopped in an ice bath. Once cooled to room temperature, 2 mL of analytical-grade toluene was added, followed by vortexing for another 20 seconds. The absorbance of the supernatant was measured at 520 nm using a spectrophotometer.

#### 2.3.4 Physiological analyses

At 60 DAT, gas exchange measurements were performed on the fourth fully expanded leaf from the plant apex at 7:00 a.m., using an infrared gas analyzer (IRGA), model CIRAS-3, under a constant light intensity of 1,800 μmol photons m^-2^ s^-1^. The following variables were assessed: stomatal conductance (*g_s_*), net CO_2_ assimilation rate (*A*), intercellular CO_2_ concentration (*C_i_*), and transpiration rate (*E*).

At the same time, relative water content (RWC) and electrolyte leakage (EL) were also evaluated. For RWC, ten 1 cm-diameter leaf discs were weighed to determine fresh mass (FM), then immersed in 20 mL of distilled water and left to stand for 12 hours. After this period, they were gently blotted and weighed again to obtain turgid mass (TM). The discs were then oven-dried at 75°C for 48 hours to determine dry mass (DM). RWC was calculated using the equation: RWC (%) = [(FM – DM)/(TM – DM)] × 100 .

For EL, five 1 cm-diameter leaf discs were immersed in 20 mL of distilled water and left to stand for 12 hours. The initial electrical conductivity of the solution (L_1_) was measured. The contents were then transferred to test tubes and heated in a water bath at 100°C for 1 hour. After cooling to room temperature, the final conductivity (L_2_) was measured. EL was calculated using the equation: $EL\;\left(\%\right)\;=\;\left(L_{1}/L_{2}\right)\;\times \;100$ .

#### 2.3.5 Growth and biomass accumulation

At 62 DAT, plant height (PH) was measured from the soil level to the tip of the tallest leaf. The plants were then separated into shoot and root systems. Both compartments were dried in a forced-air oven at 65°C for 72 hours to determine shoot (SDM) and root dry mass (RDM).

### 2.4 Statistical analyses

Experimental data were initially subjected to tests for residual normality (Shapiro–Wilk) and homogeneity of variance (Bartlett). Once the model assumptions were met, analysis of variance (ANOVA) was performed using the F-test (p ≤ 0.05). When a significant effect was detected for the silicon factor or for the silicon × potassium interaction, data were fitted to first- and/or second-degree linear regression models. Comparisons between treatments and controls were performed using Dunnett’s test (p ≤ 0.05). Statistical analyses were conducted using R software, and graphs were generated in SigmaPlot 15.0.

## 3 Results

### 3.1 Soil fertility

Analysis of variance revealed a significant interaction effect between silicon and potassium (Si × K) on substrate pH, electrical conductivity (EC), and the contents of S, Ca, Mg, K, Cu, Fe, Mn, and Zn (see **Supplementary Material**). Phosphorus and sodium contents exhibited isolated effects for the silicon and potassium factors. Additionally, the contrast between factorial treatments and the controls was significant for all evaluated variables.

**Figure 2:**
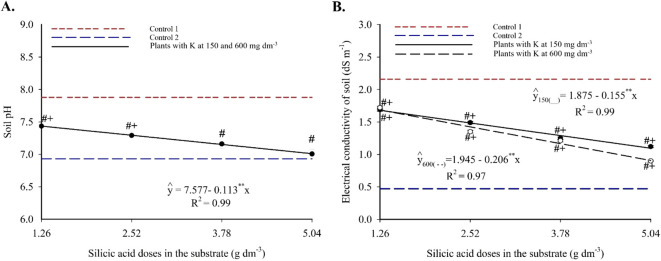
pH **(A)** and electrical conductivity **(B)** of the substrate cultivated with yellow passion fruit subjected to irrigation with saline water and applications of different doses of silicic acid and potassium. ^**^ - Values significant at 1% probability by the F test. Symbols # and + indicate significant differences compared to Control 1 (irrigated with saline water – EC 4.0 dS m^-1^) and Control 2 (irrigated with low salinity water – EC 0.5 dS m^-1^), respectively, according to Dunnett’s test. Error bars represent the standard error of the mean [**(A)** n = 10; **(B)** n = 5].

Substrate pH showed a linear decreasing trend with increasing silicic acid doses, regardless of the potassium level (**Figure 2A**). Values decreased from 7.43 to 7.00 between 1.26 and 5.04 g dm^-3^ of silicic acid, indicating a gradual acidification of the substrate. Treatments containing silicon differed significantly from both controls: pH values were lower than Control 1 (no silicon, high salinity) and higher than Control 2 (no silicon, low salinity), suggesting a moderating effect of silicon on pH.

Substrate electrical conductivity also responded linearly and negatively to silicon, with more pronounced declines at higher potassium doses (**Figure 2A**). At 150 and 600 mg dm^-3^ of K, EC decreased by 0.155 and 0.206 dS m^-1^, respectively, per unit increase in silicon. The lowest EC values observed were 1.09 and 0.906 dS m^-1^ at 5.04 g dm^-3^ of silicon. All treatments resulted in EC values between those of the controls, with statistically significant differences according to Dunnett’s test (see **Supplementary Material**), highlighting silicon’s efficacy in mitigating substrate salinity.

**Figure 3:**
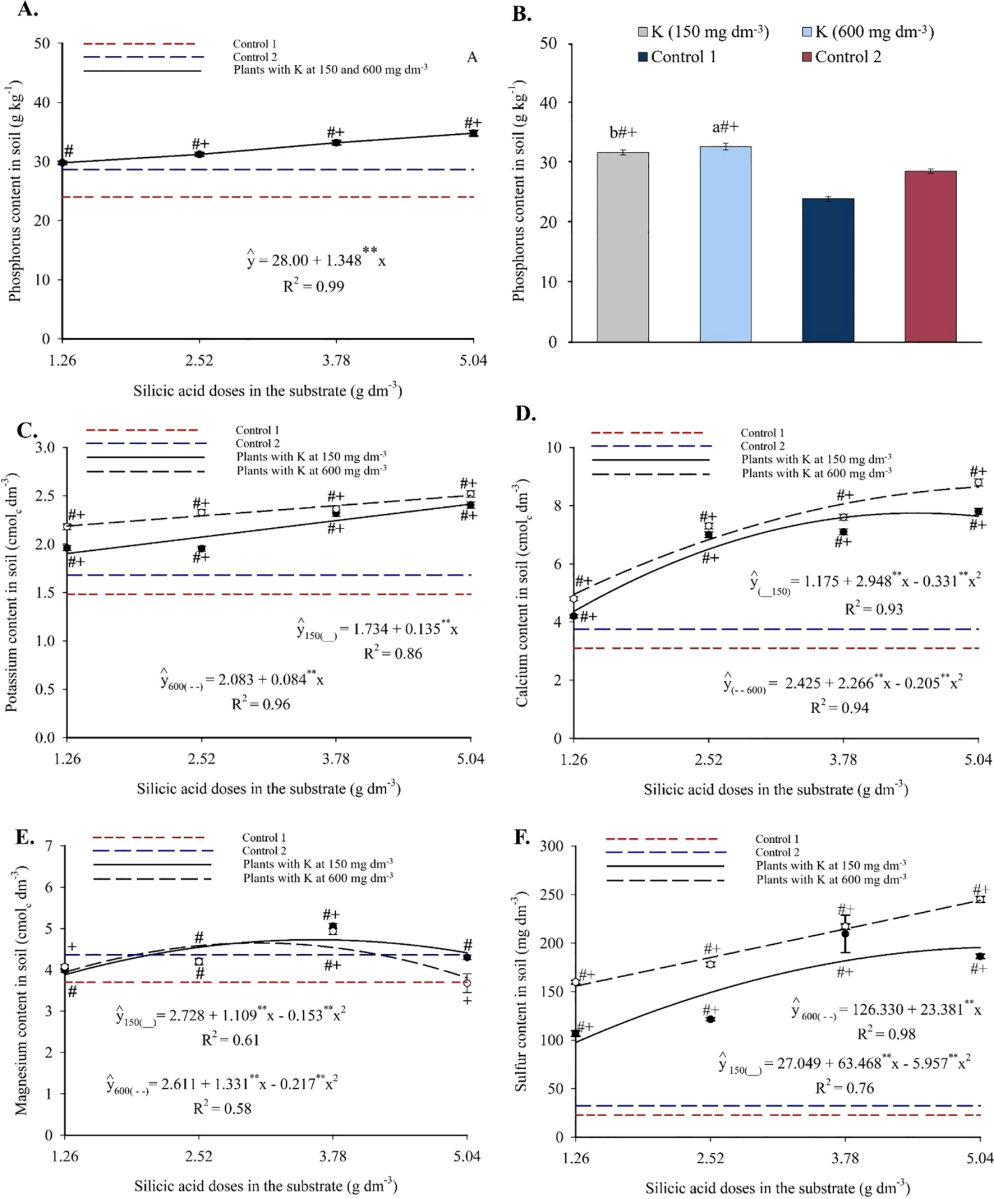
Phosphorus **(A, B)**, potassium **(C)**, calcium **(D)**, magnesium **(E)**, and sulfur **(E)** contents in the substrate cultivated with yellow passion fruit subjected to irrigation with saline water and applications of different doses of silicic acid and potassium. ^**^Values significant at 1% probability by the F test. Symbols # and + indicate significant differences compared to Control 1 (irrigated with saline water – EC 4.0 dS m^-1^) and Control 2 (irrigated with low salinity water – EC 0.5 dS m^-1^), respectively, according to Dunnett’s test. Error bars represent the standard error of the mean [**(A, C–F)** n = 5; **(B)** n = 10].

Phosphorus content in the substrate increased linearly with silicon application, reaching a maximum of 34.79 g kg^-1^ at the highest silicic acid dose (**Figure 3A**), representing an increment of 1.348 g kg^-1^ of P per unit of silicon. The two potassium doses did not differ significantly in P content, but both significantly increased available phosphorus compared to the controls (**Figure 3B**).

Potassium content increased linearly with silicon across both potassium levels (**Figure 3C**), ranging from 1.87 to 2.41 g kg^-1^ at 150 mg dm^-3^ of K, and from 2.09 to 2.51 g kg^-1^ at 600 mg dm^-3^ of K. Silicon addition enhanced K availability regardless of potassium level, and all treatments were statistically superior to the controls.

Calcium levels were also influenced by the Si × K interaction (**Figure 3D**). A progressive increase was observed up to 4.45 and 5.04 g dm^-3^ of silicon, reaching maximum Ca contents of 7.74 and 8.64 cmol_c_ dm^-3^ at 150 and 600 mg dm^-3^ of K, respectively. All treatments had significantly higher Ca levels than the controls, indicating that silicon enhanced calcium availability even under saline conditions.

The magnesium content displayed a quadratic response at both potassium levels (**Figure 3E**), with maximum values of 4.73 and 4.65 cmolc dm^-3^ observed at 3.62 and 3.07 g dm^-3^ of silicon, respectively. Intermediate silicon doses promoted greater Mg accumulation in the substrate, with several combinations significantly outperforming the controls according to Dunnett’s test (see **Supplementary Material**).

Sulfur content responded positively to silicon, fitting polynomial models (**Figure 3F**). Maximum values were 195.61 mg dm^-3^ (150 mg dm^-3^ K) and 244.17 mg dm^-3^ (600 mg dm^-3^ K), indicating a favorable interaction between silicon and potassium. These values were significantly higher than those of the controls (see **Supplementary Material**), suggesting that silicon also enhanced sulfur availability in the substrate.

**Figure 4:**
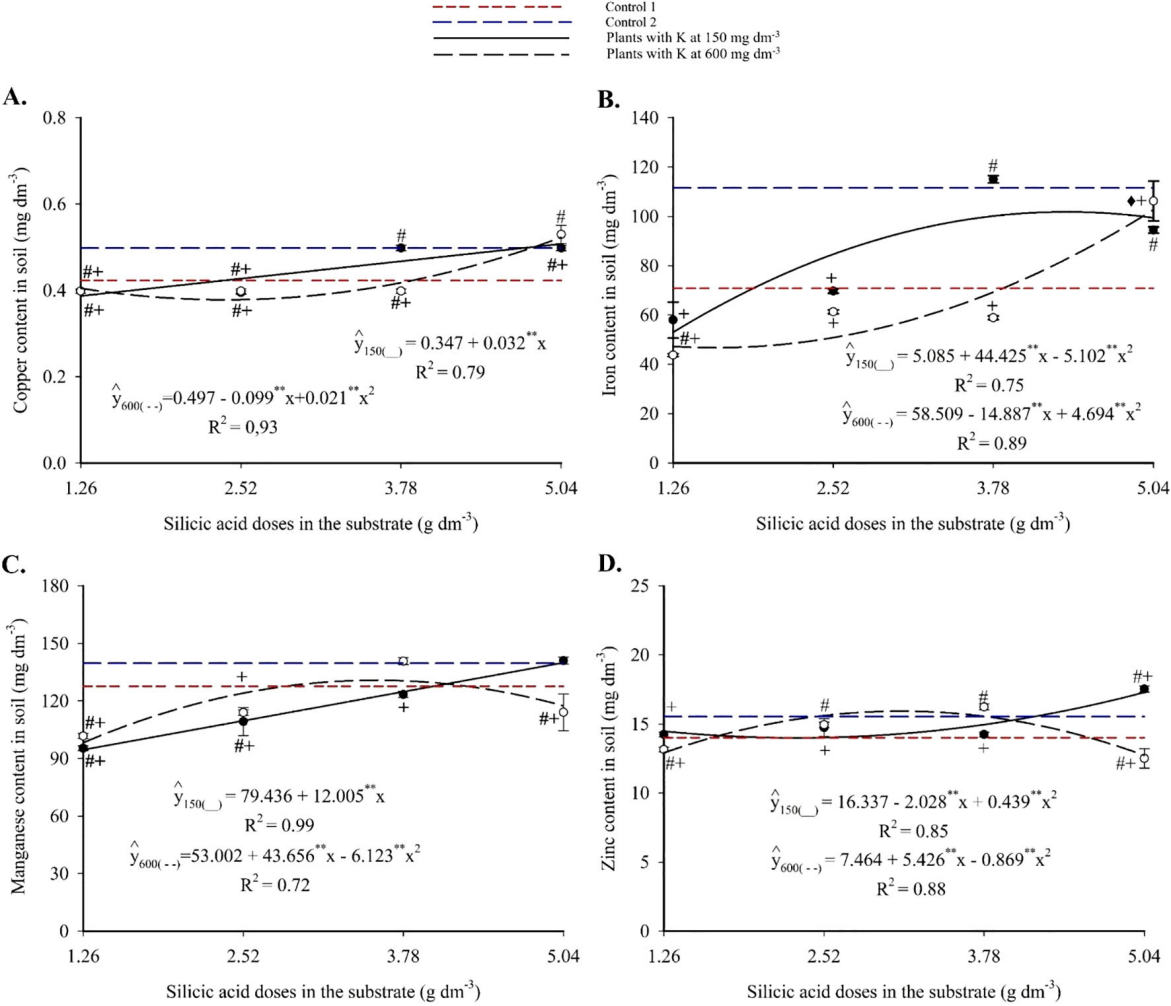
Copper **(A)**, iron **(B)**, manganese **(C)**, and zinc **(D)** contents in the substrate cultivated with yellow passion fruit subjected to irrigation with saline water and applications of different doses of silicic acid and potassium. ^**^Values significant at 1% probability by the F test. Symbols # and + indicate significant differences compared to Control 1 (irrigated with saline water – EC 4.0 dS m^-1^) and Control 2 (irrigated with low salinity water – EC 0.5 dS m^-1^), respectively, according to Dunnett’s test. Error bars represent the standard error of the mean (n = 5).

Substrate micronutrient contents showed notable responses to the treatments. Copper increased with silicon application (**Figure 4A**), exhibiting a linear trend at 150 mg dm^-3^ of K and reaching 0.51 mg dm^-3^. At 600 mg dm^-3^ of K, the response was quadratic, with a maximum of 0.53 mg dm^-3^. Comparisons with the controls revealed statistically significant differences, with higher Cu accumulation under silicon-treated conditions.

Iron exhibited a quadratic response to silicon fertilization, with a maximum of 102.71 mg dm^-3^ at 5.04 g dm^-3^ of silicon and 600 mg dm^-3^ of K (**Figure 4B**). At 150 mg dm^-3^ of K, maximum Fe values were lower but still significantly higher than those in the controls, as confirmed by Dunnett’s test (see **Supplementary Material**). Manganese content increased linearly with silicon at 150 mg dm^-3^ of K, peaking at 139.94 mg dm^-3^ (**Figure 4C**). At 600 mg dm^-3^ of K, a quadratic trend was observed, with a maximum of 130.82 mg dm^-3^. Intermediate silicon doses resulted in significantly higher Mn levels compared to the controls. Zinc content also followed a quadratic trend, with maximum values of 17.26 and 15.92 mg dm^-3^ at 4.21 and 4.59 g dm^-3^ of silicon, respectively, for the 150 and 600 mg dm^-3^ K treatments (**Figure 4D**). Statistical analysis confirmed that silicon-containing treatments significantly increased Zn levels relative to the controls.

**Figure 5:**
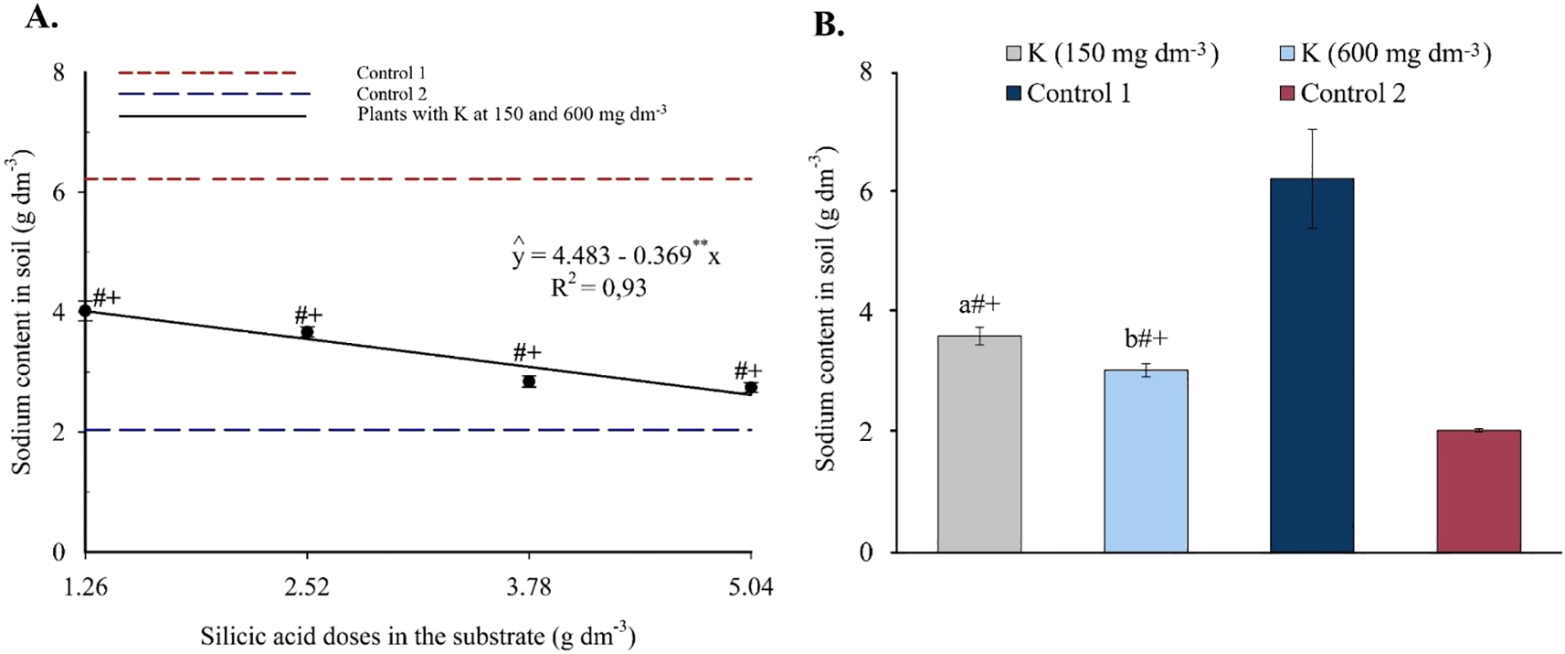
Sodium content in the substrate as a function of silicic acid doses **(A)** and potassium doses **(B)**. ^**^Values significant at 1% probability by the F test. Symbols # and + indicate significant differences compared to Control 1 (irrigated with saline water – EC 4.0 dS m^-1^) and Control 2 (irrigated with low salinity water – EC 0.5 dS m^-1^), respectively, according to Dunnett’s test. Error bars represent the standard error of the mean (n = 10).

Finally, substrate sodium content exhibited a linear decreasing response to silicon, ranging from 4.01 to 2.62 cmolc dm^-3^ as silicic acid doses increased (**Figure 5A**). With potassium applied independently (**Figure 5B**), both levels (150 and 600 mg dm^-3^) significantly reduced exchangeable Na^+^ compared to Control 1, which was irrigated with saline water.

### 3.2 Foliar mineral element content

The analysis of variance (see **Supplementary Material**) indicated a significant interaction effect (p ≤ 0.05) between silicon and potassium on the foliar concentrations of all evaluated elements: N, P, K, Ca, Mg, S, Na, Cu, Fe, Mn, Zn, and Si. Contrasts between the factorial treatments and the controls (Control 1 - saline water; and Control 2 - non-saline water) were also significant for all variables, highlighting the influence of water salinity and silicon and potassium fertilization on plant nutrition.

**Figure 6:**
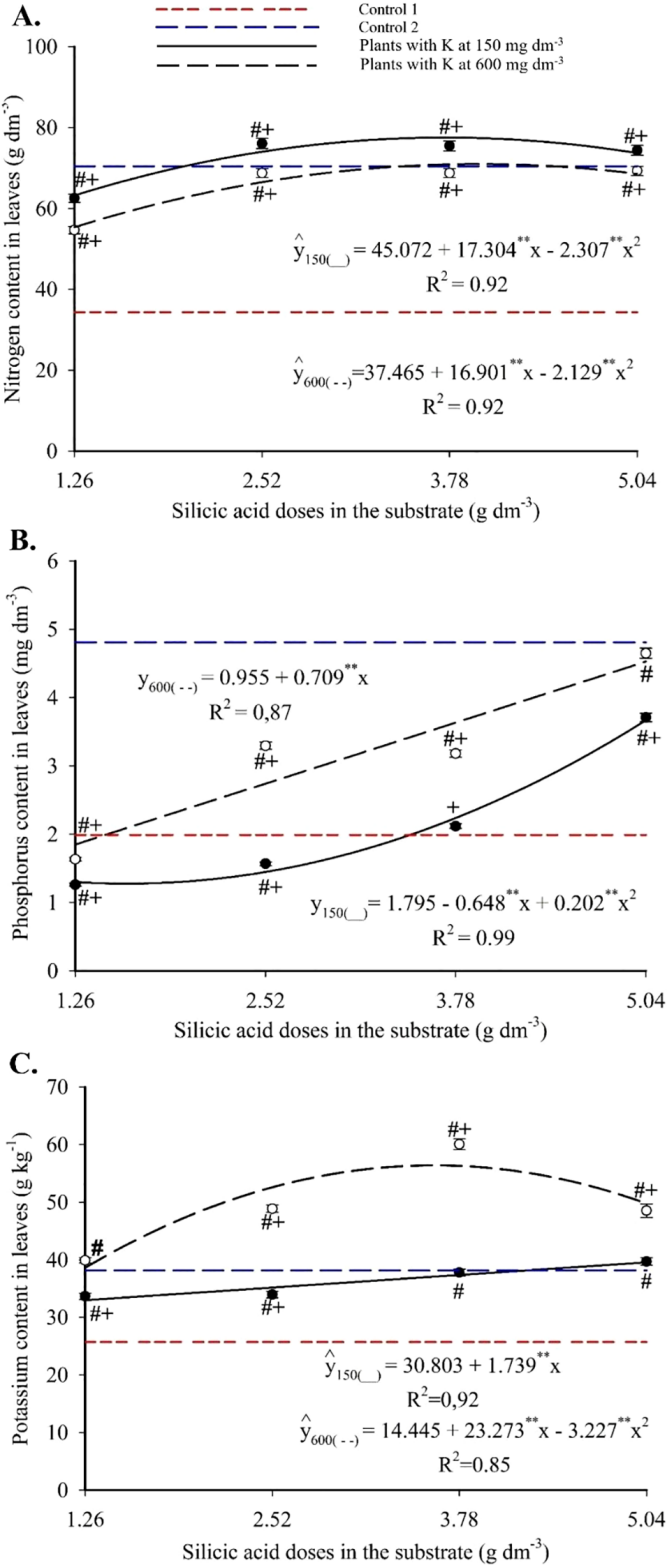
Nitrogen **(A)**, phosphorus **(B)** and potassium **(C)** contents in the leaves of passion fruit subjected to irrigation with saline water and applications of different doses of silicic acid and potassium. ^**^Values significant at 1% probability by the F test. Symbols # and + indicate significant differences compared to Control 1 (irrigated with saline water – EC 4.0 dS m^-1^) and Control 2 (irrigated with low salinity water – EC 0.5 dS m^-1^), respectively, according to Dunnett’s test. Error bars represent the standard error of the mean (n = 5).

Foliar nitrogen content followed a quadratic response to silicon doses. The combinations of 3.75 g dm^-3^ of Si with 150 mg dm^-3^ of K, and 3.97 g dm^-3^ of Si with 600 mg dm^-3^ of K resulted in the highest N concentrations, 77.52 and 71.01 g kg^-1^, respectively (**Figure 6A**). These values exceeded those of Control 1 in all treatments and were either superior or statistically equivalent to Control 2, except at the lowest Si dose.

Foliar phosphorus exhibited a quadratic response with 150 mg dm^-3^ of K, ranging from 1.27 to 3.66 g kg^-1^ and peaking at 5.04 g kg^-1^ with 5.04 g dm^-3^ of Si. With 600 mg dm^-3^ of K, a linear increase was observed, with an average increment of 0.71 g kg^-1^ of P per g dm^-3^ of Si, reaching 4.53 g kg^-1^ (**Figure 6B**). Foliar potassium increased linearly with Si at 150 mg dm^-3^ of K, reaching 39.57 g kg^-1^, and followed a quadratic model at 600 mg dm^-3^ of K, peaking at 56.41 g kg^-1^ at 3.61 g dm^-3^ of Si (**Figure 6C**). At both K levels, all treatments surpassed Control 1, and those with higher K doses also exceeded Control 2.

**Figure 7:**
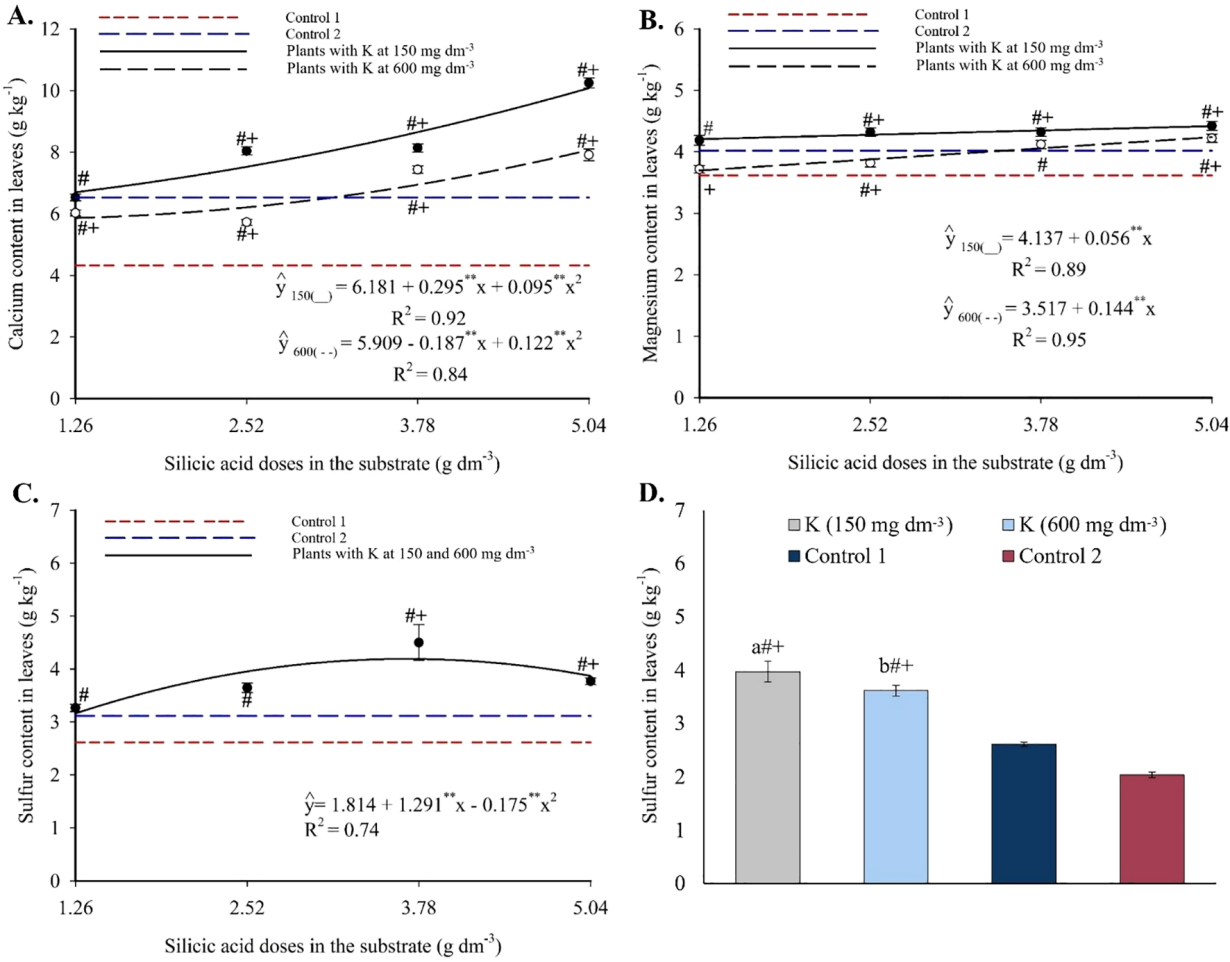
Calcium **(A)**, magnesium **(B)**, and sulfur **(C, D)** contents in the leaves of passion fruit subjected to irrigation with saline water and applications of different doses of silicic acid and potassium. ^**^Values significant at 1% probability by the F test. Symbols # and + indicate significant differences compared to Control 1 (irrigated with saline water – EC 4.0 dS m^-1^) and Control 2 (irrigated with low salinity water – EC 0.5 dS m^-1^), respectively, according to Dunnett’s test. Error bars represent the standard error of the mean [**(A, B)** n = 5; **(C, D)** n = 10].

Calcium concentrations followed a quadratic trend in response to Si (**Figure 7A**), with minimum values between 5.87 and 6.87 g kg^-1^ at intermediate doses and maximum values of 10.08 g kg^-1^ (150 mg dm^-3^ K) and 8.07 g kg^-1^ (600 mg dm^-3^ K) at 5.04 g dm^-3^ of Si. Most combinations produced values higher than those of the controls.

Foliar magnesium showed a linear increase with silicon (**Figure 7B**). At 150 mg dm^-3^ of K, Mg ranged from 3.70 to 4.21 g kg^-1^, with an increase of 0.056 g kg^-1^ per Si unit. At 600 mg dm^-3^ of K, Mg varied from 4.24 to 4.42 g kg^-1^, with a 0.144 g kg^-1^ increment per Si unit. At both K levels, higher Si doses exceeded Control 1, and most treatments also surpassed Control 2.

Foliar sulfur followed a quadratic model in response to Si (**Figure 7C**), with a maximum of 4.19 g kg^-1^ at 3.69 g dm^-3^ of Si, regardless of K dose. Control values ranged from 2.61 to 3.12 g kg^-1^ and were significantly lower than those of the Si treatments. Both K levels also increased foliar S relative to the controls (**Figure 7D**).

**Figure 8:**
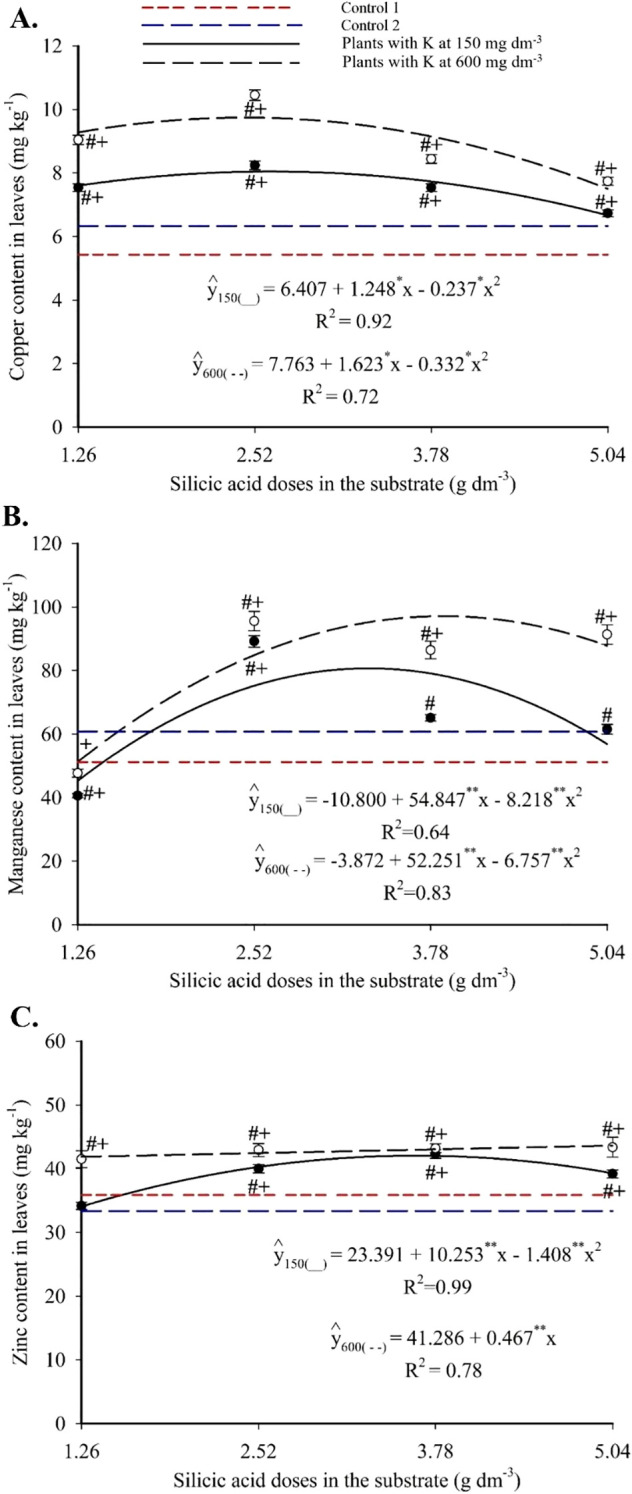
Copper **(A)**, manganese **(B)**, and zinc **(C)** contents in the leaves of passion fruit subjected to irrigation with saline water and applications of different doses of silicic acid and potassium. ^**^Values significant at 1% probability by the F test. Symbols # and + indicate significant differences compared to Control 1 (irrigated with saline water – EC 4.0 dS m^-1^) and Control 2 (irrigated with low salinity water – EC 0.5 dS m^-1^), respectively, according to Dunnett’s test. Error bars represent the standard error of the mean (n = 5).

Copper showed a quadratic response to Si, with maxima of 5.42 mg kg^-1^ (2.63 g dm^-3^ Si + 150 mg dm^-3^ K) and 6.33 mg kg^-1^ (2.44 g dm^-3^ Si + 600 mg dm^-3^ K) (**Figure 8A**). These peak values exceeded those of both controls. Foliar manganese followed a quadratic pattern (**Figure 8B**), with maximum of 80.71 mg kg^-1^ (3.34 g dm^-3^ Si + 150 mg dm^-3^ K) and 97.14 mg kg^-1^ (3.87 g dm^-3^ Si + 600 mg dm^-3^ K). Most treatments yielded higher values than the controls. Zinc also exhibited a quadratic response, with peak values of 42.06 mg kg^-1^ (3.64 g dm^-3^ Si + 150 mg dm^-3^ K) and 43.64 mg kg^-1^ (5.04 g dm^-3^ Si + 600 mg dm^-3^ K), with nearly all treatments exceeding the control values (**Figure 8C**).

**Figure 9:**
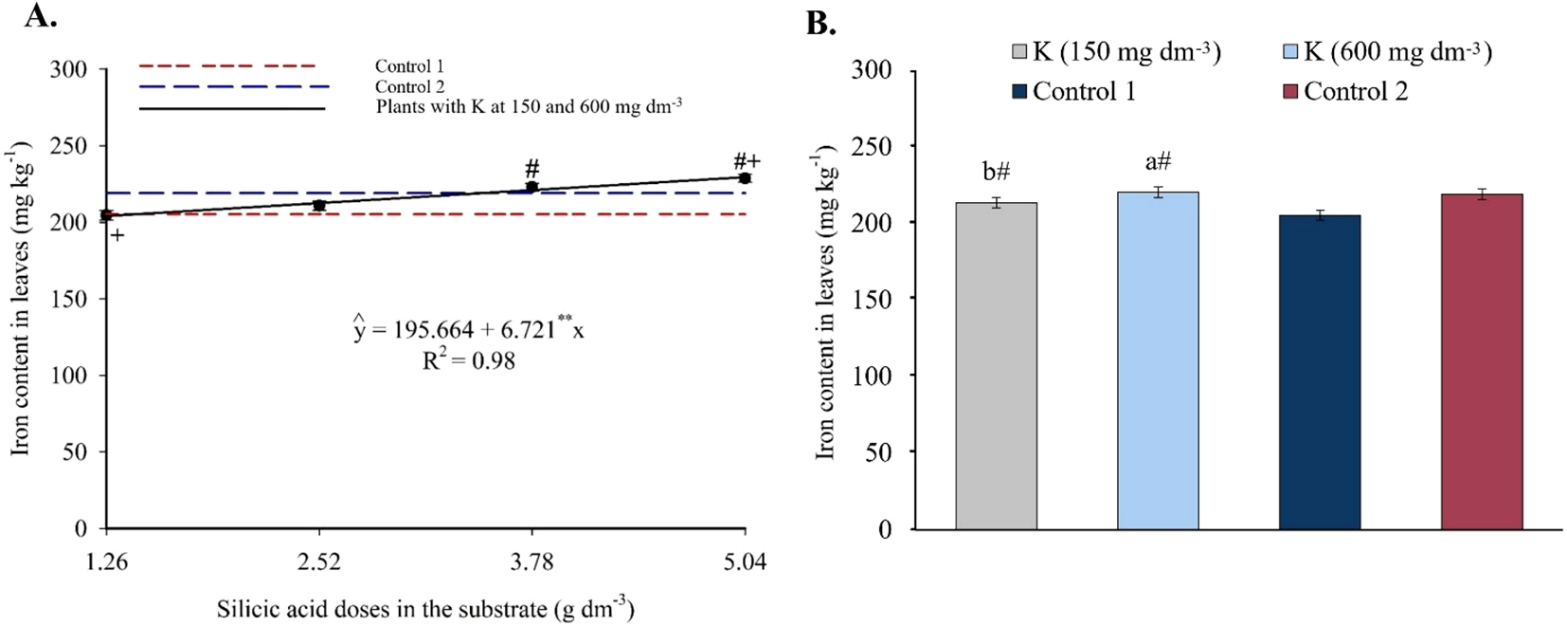
Iron content in the leaves of passion fruit as a function of silicic acid doses **(A)** and potassium doses **(B)**. ^**^Values significant at 1% probability by the F test. Symbols # and + indicate significant differences compared to Control 1 (irrigated with saline water – EC 4.0 dS m^-1^) and Control 2 (irrigated with low salinity water – EC 0.5 dS m^-1^), respectively, according to Dunnett’s test. Error bars represent the standard error of the mean (n = 10).

Foliar iron content increased linearly with silicon, reaching 229.54 mg kg^-1^ at 5.04 g dm^-3^ of Si (**Figure 9A**). Doses of 3.78 and 5.04 g dm^-3^ yielded significantly higher values than the controls. Under saline irrigation (EC 4.0 dS m^-1^), Fe^2+^ levels were higher than in Control 1. Compared to Control 2 (non-saline water), Fe^2+^ contents were similar at both K levels (**Figure 9B**).

**Figure 10:**
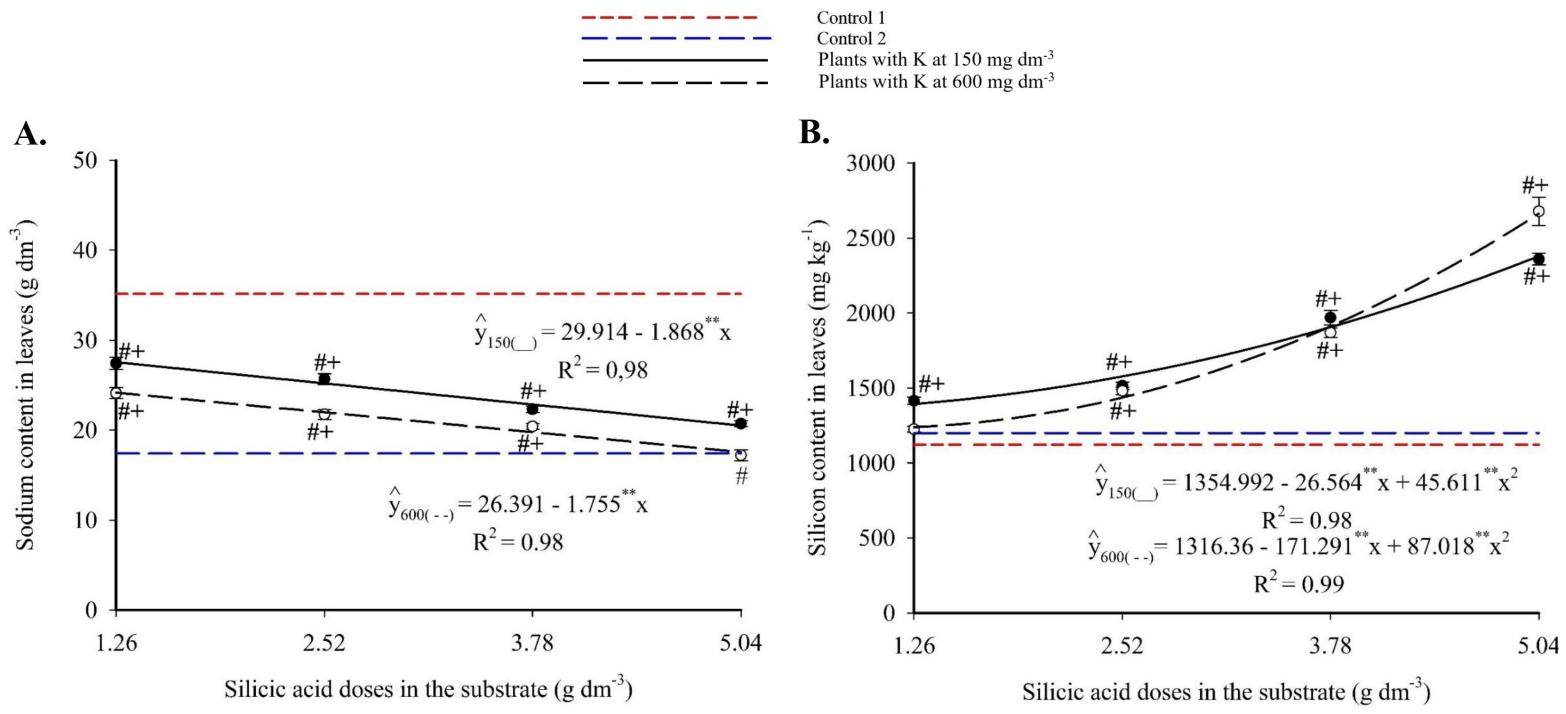
Sodium **(A)**, and silicon **(C)** contents in the leaves of passion fruit subjected to irrigation with saline water and applications of different doses of silicic acid and potassium. ^**^Values significant at 1% probability by the F test. Symbols # and + indicate significant differences compared to Control 1 (irrigated with saline water – EC 4.0 dS m^-1^) and Control 2 (irrigated with low salinity water – EC 0.5 dS m^-1^), respectively, according to Dunnett’s test. Error bars represent the standard error of the mean (n = 5).

Foliar sodium showed a decreasing linear trend with increasing Si (**Figure 10A**), ranging from 27.56-25.43 g kg^-1^ to 20.49-22.59 g kg^-1^ for 150 and 600 mg dm^-3^ of K, respectively. These reductions represented up to 34.5% less than Control 1. At the highest Si and K doses, Na levels matched those of Control 2.

Foliar silicon followed a quadratic response (**Figure 10B**), with maxima of 2,379.70 mg kg^-1^ (150 mg dm^-3^ K) and 2,663.45 mg kg^-1^ (600 mg dm^-3^ K), both at 5.04 g dm^-3^ Si. All combinations produced significantly higher Si levels than both Control 1 and Control 2 (1,121.86 and 1,199.02 mg kg^-1^, respectively).

On average, the accumulation order of foliar mineral elements was: N > K > Na > Ca > S > Mg > P > Si > Fe > Mn > Zn > Cu.

### 3.3 Biochemical characteristics

Analysis of variance (see **Supplementary Material**) revealed a significant interaction (p ≤ 0.05) between silicon and potassium on foliar proline content. Individually, silicon and potassium had significant effects on total chlorophyll concentration. Statistically significant differences were also observed between the treatments and the controls for all these variables.

**Figure 11:**
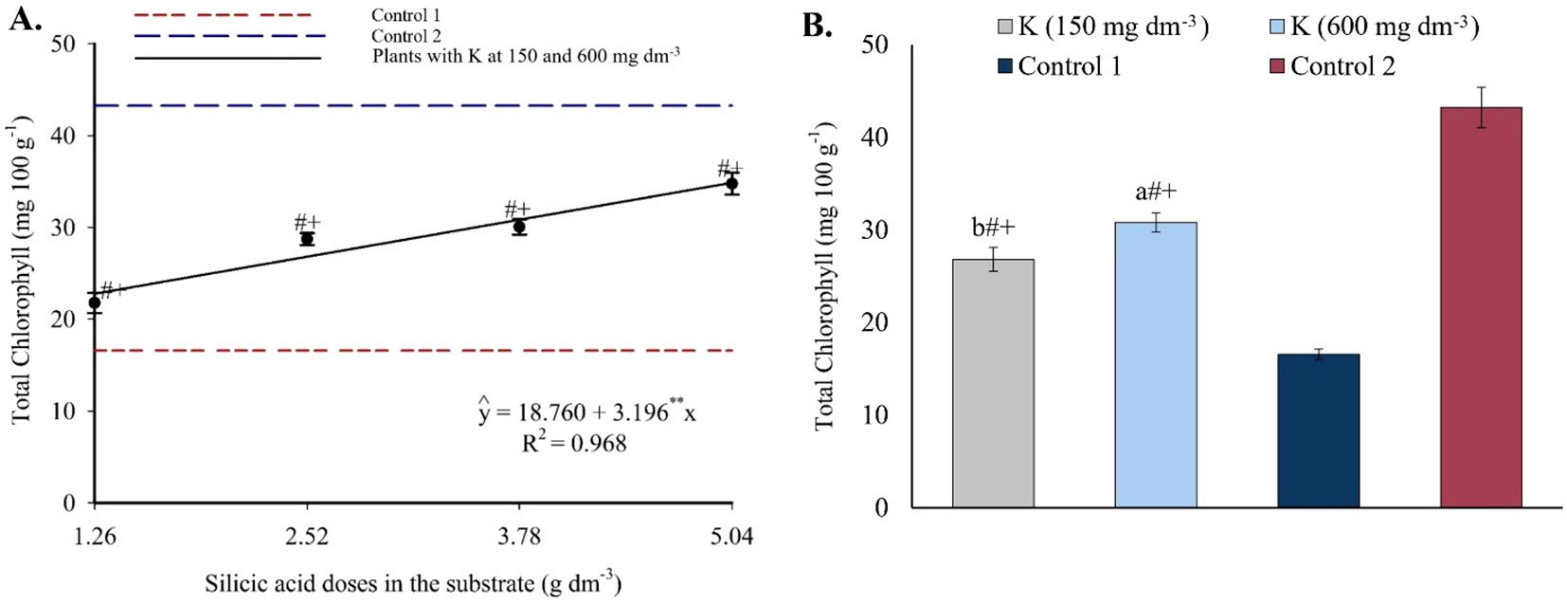
Total chlorophyll content in the leaves of passion fruit as a function of silicic acid doses **(A)** and potassium doses **(B)**. ^**^Values significant at 1% probability by the F test. Symbols # and + indicate significant differences compared to Control 1 (irrigated with saline water – EC 4.0 dS m^-1^) and Control 2 (irrigated with low salinity water – EC 0.5 dS m^-1^), respectively, according to Dunnett’s test. Error bars represent the standard error of the mean (n = 10).

Total chlorophyll increased with higher Si doses (**Figure 11A**). Values ranged from 22.79 to 34.87 mg 100 g^-1^ across the Si range of 1.26 to 5.04 g dm^-3^. In potassium treatments, total chlorophyll levels were higher than in Control 1 and lower than in Control 2, with higher values observed under 600 mg dm^-3^ of K (**Figure 11B**).

**Figure 12:**
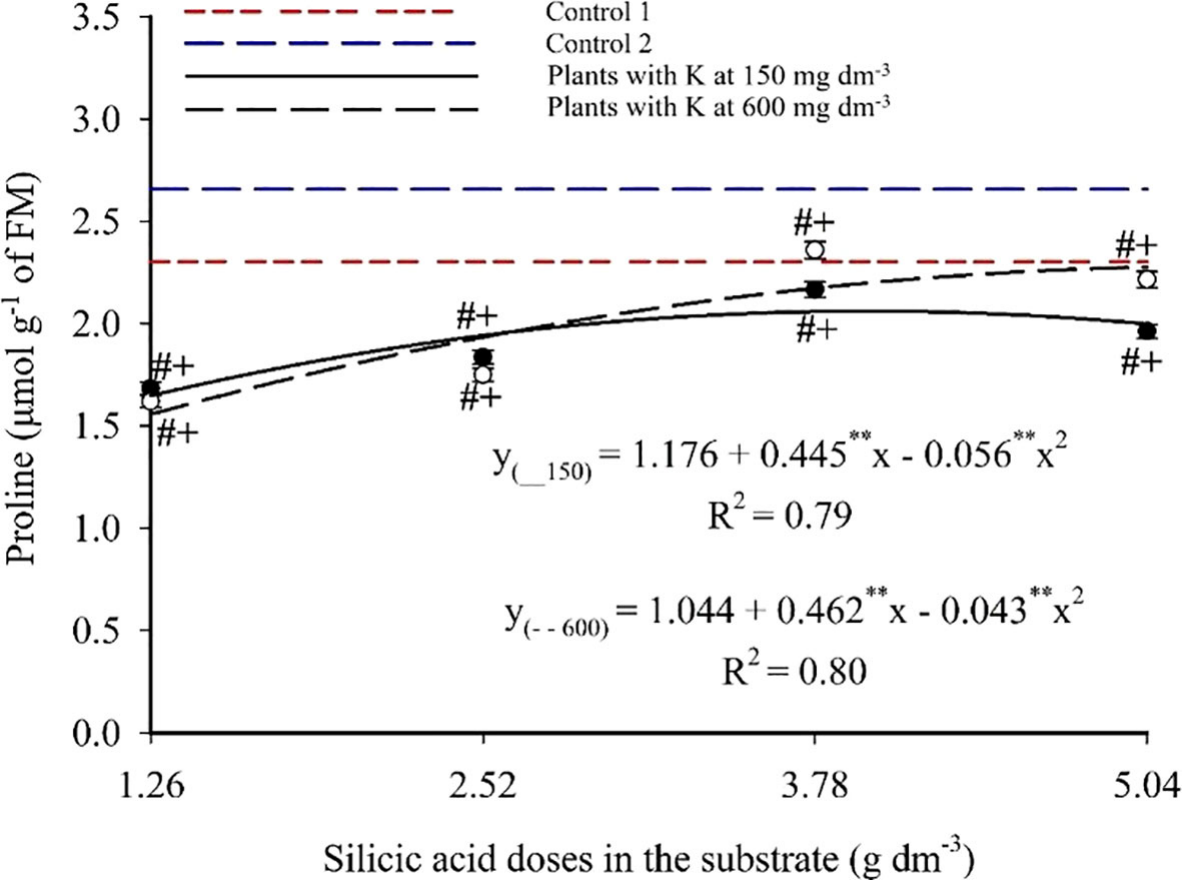
Proline content in the leaves of passion fruit subjected to irrigation with saline water and applications of different doses of silicic acid and potassium. ^**^Values significant at 1% probability by the F test. Symbols # and + indicate significant differences compared to Control 1 (irrigated with saline water – EC 4.0 dS m^-1^) and Control 2 (irrigated with low salinity water – EC 0.5 dS m^-1^), respectively, according to Dunnett’s test. Error bars represent the standard error of the mean (n = 5).

Foliar proline content fits a second-degree polynomial model (**Figure 12**), with maxima of 2.06 and 2.28 mg 100 g^-1^ at 3.97 and 5.04 g dm^-3^ of Si combined with 150 and 600 mg dm^-3^ of K, respectively. Minimum values of 1.65 and 1.56 mg 100 g^-1^ were observed at the lowest Si dose (1.26 g dm^-3^), corresponding to increases of 24.85% and 46.15%. Si treatments resulted in lower proline levels compared to Controls 1 (2.30 mg 100 g^-1^) and 2 (2.66 mg 100 g^-1^), with statistically significant group differences.

### 3.4 Physiological characteristics

The analysis of variance (see **Supplementary Material**) revealed significant effects (p ≤ 0.05) of silicon and potassium on stomatal conductance, CO_2_ assimilation rate, intercellular CO_2_ concentration, transpiration rate, relative water content, and electrolyte leakage. The silicon × potassium interaction was significant only for *E*. Significant differences were also observed between treatments and controls for all evaluated variables.

**Figure 13:**
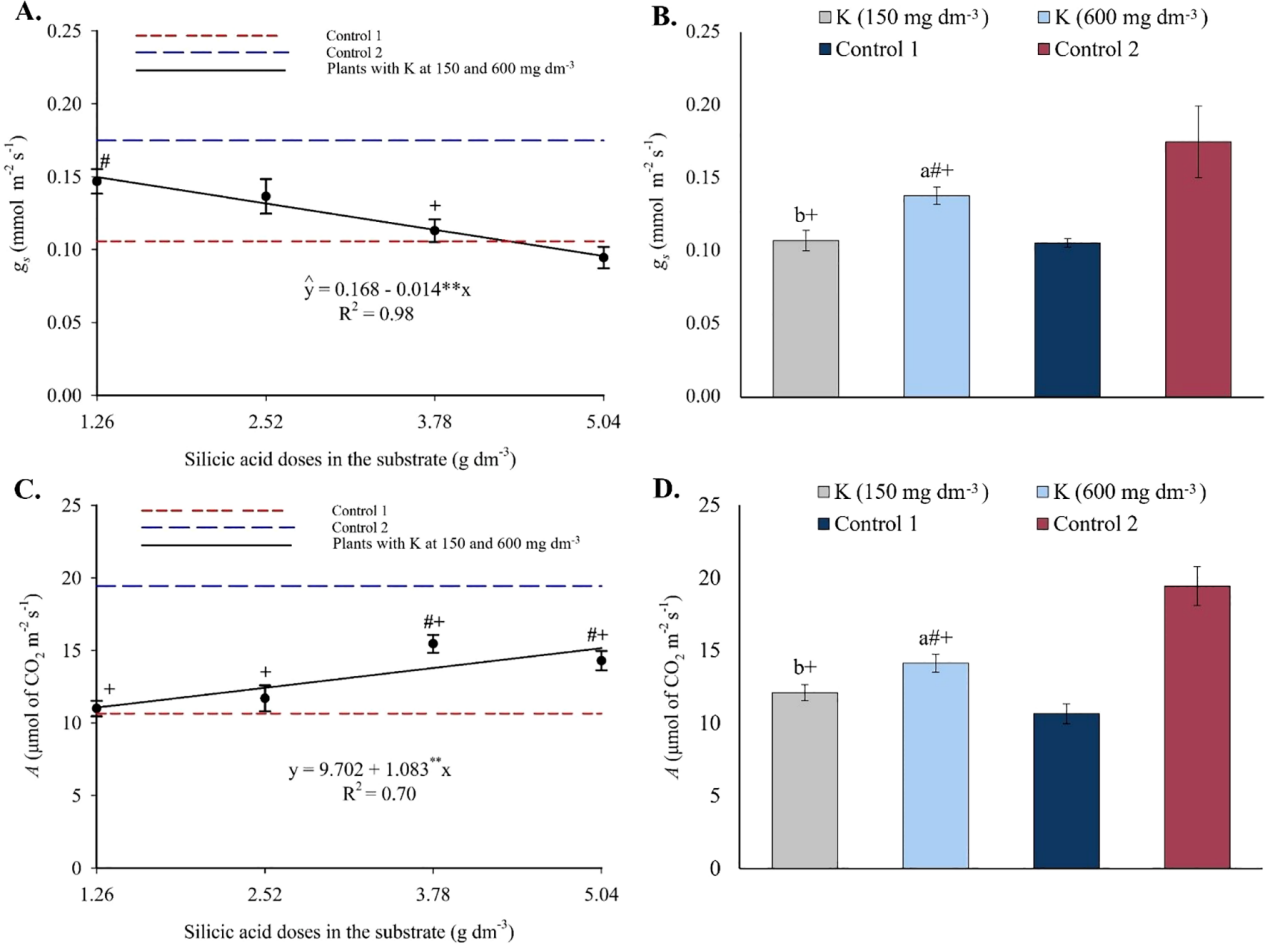
Stomatal conductance – *g_s_* **(A, B)**, and CO_2_ assimilation rate – *A* **(C, D)**, in the leaves of passion fruit as a function of silicic acid doses and potassium doses. ^**^Values significant at 1% probability by the F test. Symbols # and + indicate significant differences compared to Control 1 (irrigated with saline water – EC 4.0 dS m^-1^) and Control 2 (irrigated with low salinity water – EC 0.5 dS m^-1^), respectively, according to Dunnett’s test. Error bars represent the standard error of the mean (n = 10).

Stomatal conductance decreased progressively with increasing silicon doses, fitting a linear regression model (**Figure 13A**). Values observed in treatments were higher than in control 1 but lower than in control 2. Regarding potassium, only the highest dose increased *g_s_* values (**Figure 13B**). CO_2_ assimilation rate increased linearly with rising silicon levels (**Figure 13C**), exceeding values observed in control 1 but remaining below those of control 2. Potassium fertilization also increased *A*, but only at the highest dose (**Figure 13D**).

**Figure 14:**
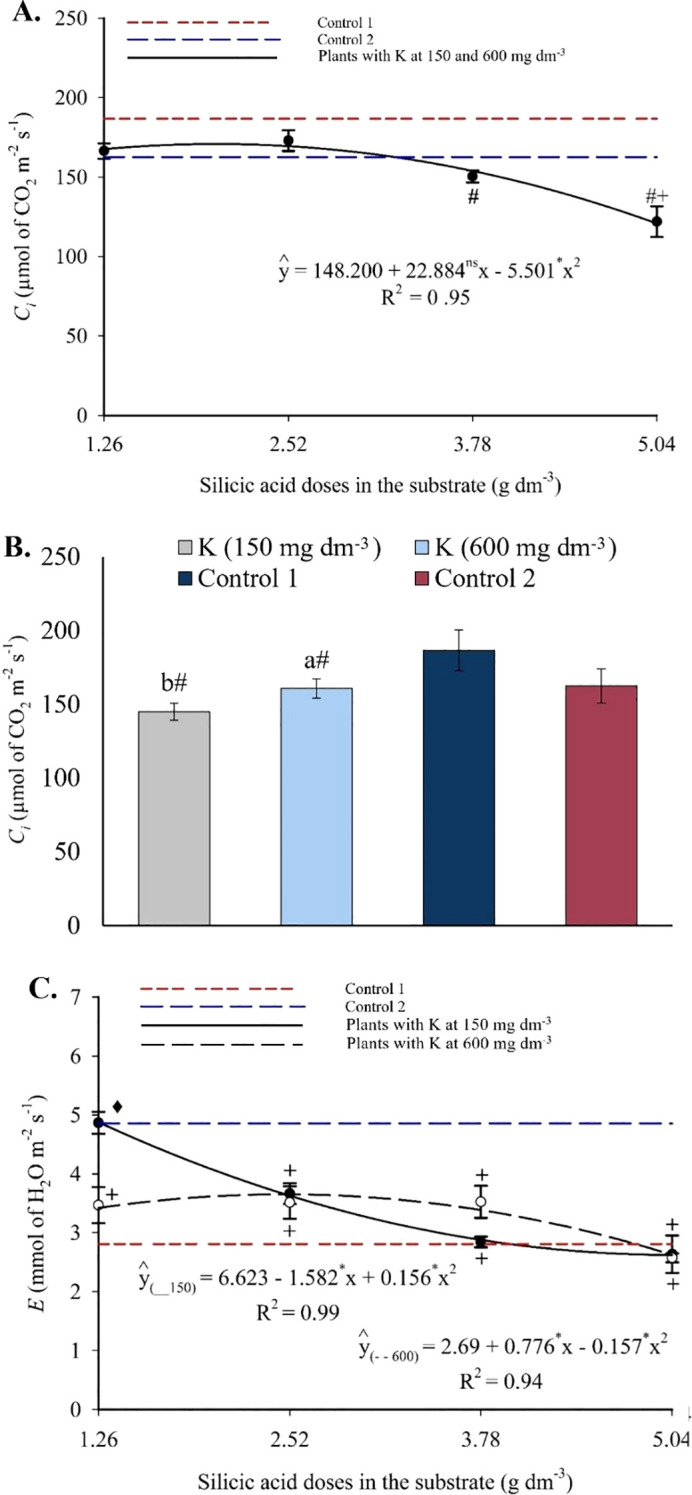
Intercellular CO_2_ concentration – *C_i_* **(A, B)**, and transpiration – *E* **(C)** in the leaves of passion fruit as a function of silicic acid doses and potassium doses. ^**^Values significant at 1% probability by the F test. Symbols # and + indicate significant differences compared to Control 1 (irrigated with saline water – EC 4.0 dS m^-1^) and Control 2 (irrigated with low salinity water – EC 0.5 dS m^-1^), respectively, according to Dunnett’s test (p < 0.05). Error bars represent the standard error of the mean [**(A, B)** n = 10; **(C)** n = 10].

Intercellular CO_2_ concentration followed a quadratic trend in response to silicon, with reductions at higher doses (**Figure 14A**). Treatments with silicon showed lower *C_i_* values than both controls. Potassium application also contributed to reductions in *C_i_* compared to control 1 (**Figure 14B**). The transpiration rate was influenced by the silicon × potassium interaction and followed a quadratic pattern (**Figure 14C**). At 150 mg dm^-3^ of potassium, *E* was highest (4.88 μmol CO_2_ m^-2^ s^-1^) at the lowest silicon dose and lowest (2.61 μmol CO_2_ m^-2^ s^-1^) at the highest. At 600 mg dm^-3^ of potassium, the maximum *E* (3.65 μmol CO_2_ m^-2^ s^-1^) occurred at 2.47 g dm^-3^ of silicon, decreasing to 2.61 μmol CO_2_ m^-2^ s^-1^ at the highest Si dose.

**Figure 15:**
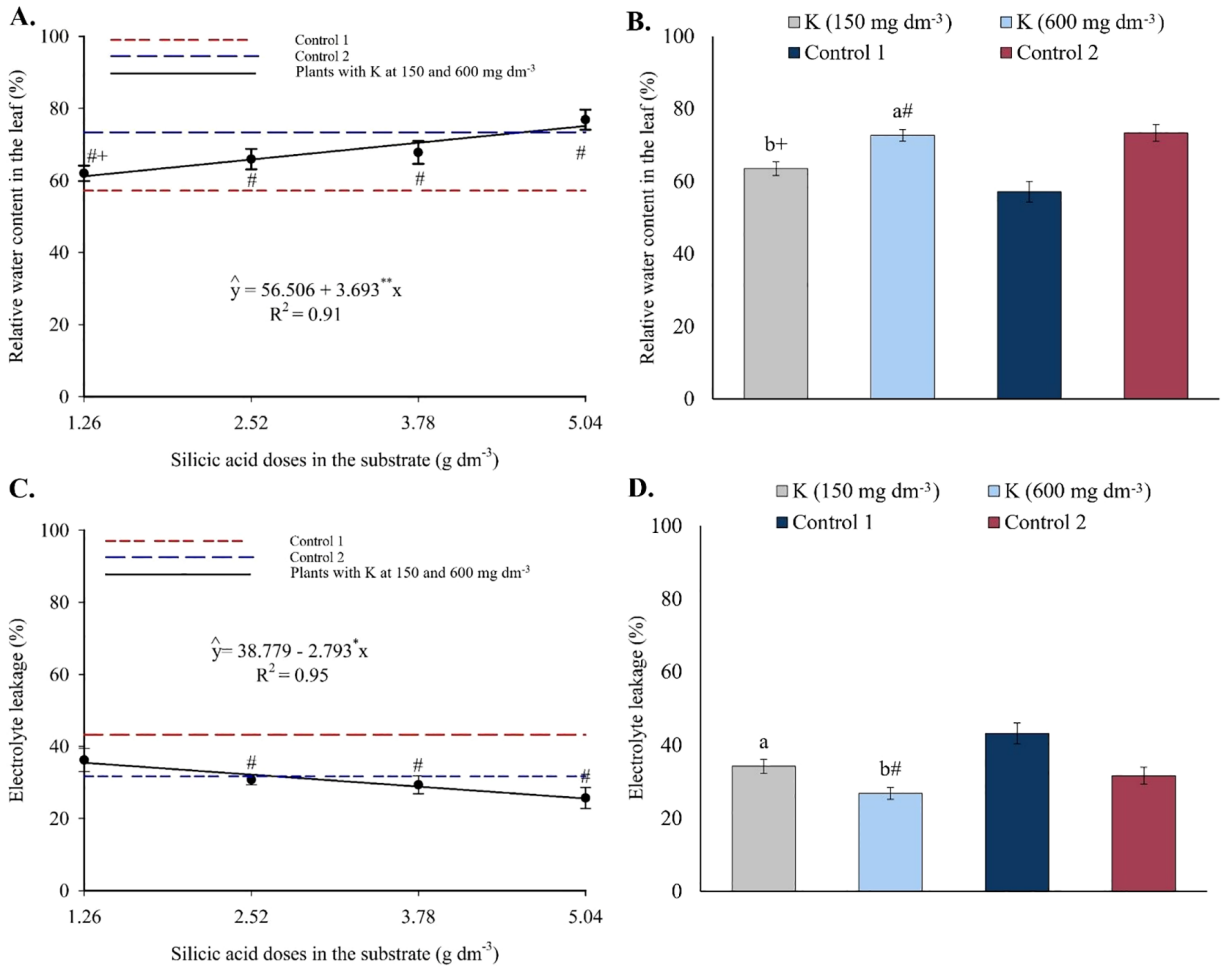
Relative water content **(A, B)** and electrolyte leakage **(C, D)** in the leaves of passion fruit as a function of silicic acid doses **(A)** and potassium doses **(B)**. ^**^Values significant at 1% probability by the F test. Symbols # and + indicate significant differences compared to Control 1 (irrigated with saline water – EC 4.0 dS m^-1^) and Control 2 (irrigated with low salinity water – EC 0.5 dS m^-1^), respectively, according to Dunnett’s test. Error bars represent the standard error of the mean (n = 10).

Relative water content increased linearly with silicon doses (**Figure 15A**), with an estimated increment of 3.69% per unit of Si applied. Regarding potassium (**Figure 15B**), only the highest dose (600 mg dm^-3^) resulted in RWC values higher than those in control 1. Electrolyte leakage showed a linear decreasing trend with increasing silicon (**Figure 15C**), dropping from 35.26% to 24.70% between the minimum and maximum doses. For potassium, EL values were higher at 150 mg dm^-3^ and lower at 600 mg dm^-3^ (**Figure 15D**), with the latter being statistically lower than control 1 and similar to control 2.

### 3.5 Growth and biomass accumulation

Significant effects of the individual factors silicon and potassium were observed on plant height (PH), shoot dry mass, and root dry mass of yellow passion fruit seedlings (see ANOVA, in **Supplementary Material**). Significant differences were also found between treatments and controls for SDM and RDM. See **Supplementary Figure** for the main phenotypic results.

**Figure 16:**
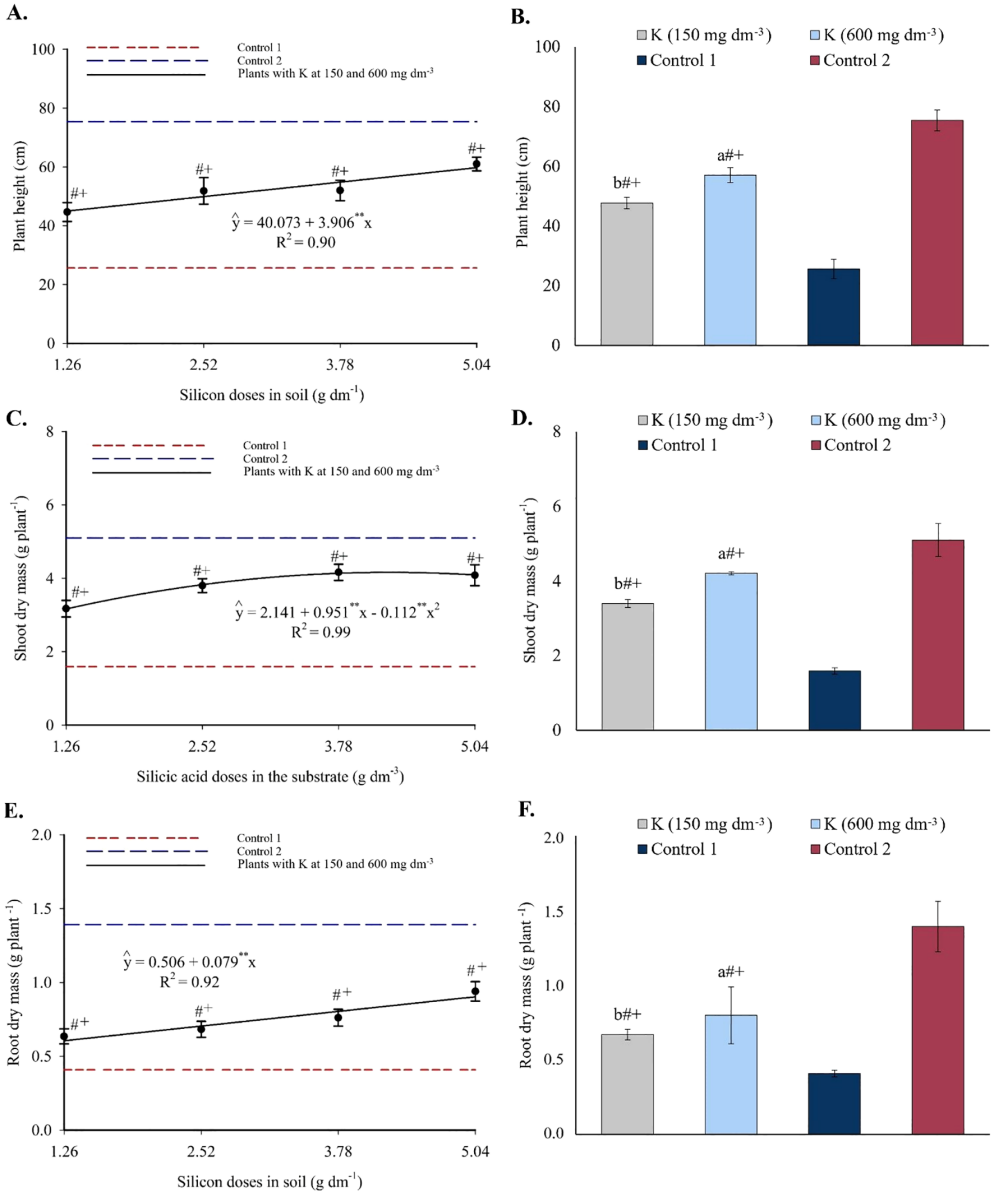
Plant height **(A, B)**, shoot dry mass **(C, D)**, and root dry mass **(E, F)** of passion fruit seedlings as a function of silicic acid doses and potassium doses. ^**^Values significant at 1% probability by the F test. Symbols # and + indicate significant differences compared to Control 1 (irrigated with saline water – EC 4.0 dS m^-1^) and Control 2 (irrigated with low salinity water – EC 0.5 dS m^-1^), respectively, according to Dunnett’s test. Error bars represent the standard error of the mean (n = 10).

Plant height increased linearly with silicon doses, reaching a maximum of 59.76 cm at the highest dose, representing a 32.82% increase from the lowest dose (**Figure 16A**). Compared to control 1, this represented a 133.44% increase. All silicon treatments resulted in plant height values lower than those of control 2. Potassium doses also promoted height increases, reaching 47.69 cm and 57.06 cm at 150 and 600 mg dm^-3^, respectively, both greater than control 1 and lower than control 2 (**Figure 16B**).

SDM followed a second-degree polynomial model, with a maximum estimated value of 4.16 g plant^-1^ at 4.25 g dm^-3^ of silicon (**Figure 16C**). This represented a 161.64% increase compared to control 1. Treatment values were still lower than those of control 2. Potassium doses resulted in SDM values of 3.40 g and 4.21 g for 150 and 600 mg dm^-3^, respectively, both higher than control 1 and lower than control 2 (**Figure 16D**).

RDM increased linearly with silicon doses, with an increment of 0.079 g per unit of Si, reaching 0.90 g at the highest dose (**Figure 16E**). This corresponds to a 119.51% increase relative to control 1. Control 2 still showed higher RDM than all treatments. Potassium application led to RDM values of 0.67 g and 0.80 g for 150 and 600 mg dm^-3^, respectively, again higher than control 1 but lower than control 2 (**Figure 16F**).

## 4 Discussion

Overall, the application of silicon, in combination with either the lowest or highest potassium dose, promoted significant changes in soil chemical properties and in the physiology of yellow passion fruit seedlings under saline stress. The results of this study demonstrate that these elements act synergistically to mitigate the effects of salinity stress in yellow passion fruit seedlings irrigated with water of electrical conductivity 4.0 dS m^-1^. Silicon contributed to improving the chemical conditions of the substrate, particularly by reducing electrical conductivity and pH (**Figure 2**), indicating a corrective effect on salinization and alkalinization induced by saline irrigation.

The linear reduction in substrate pH of plants irrigated with saline water, in response to increasing silicon doses (**Figure 2A**), is a complex phenomenon whose mechanisms are not yet fully understood. Sirisuntornlak et al. (2021) report that in various contexts, the interaction between silicon and substrate pH tends to be non-significant, suggesting the feasibility of silicon application across a wide pH range. However, Szulc et al. (2015) highlight that pH is one of the main factors regulating the availability of exchangeable silicon in soil, being influenced by properties such as texture, organic matter content, temperature, and the presence of other ions in solution.

In the present study, the average pH values observed in treatments with the highest silicon doses fell within the optimal range of 6.0 to 7.0, which is agronomically favorable for plant nutrition under saline conditions (Neina, 2019). Although not directly measured in this study, it is hypothesized that the combined application of silicon and potassium may have favored the replacement of H^+^ by K^+^ at cation exchange sites, potentially contributing to shifts in the substrate’s acid-base balance and localized proton release into the soil solution.

The decline in substrate electrical conductivity in response to silicon doses combined with both potassium sulfate levels (**Figure 2B**) highlights the role of silicon in reducing the accumulation of soluble salts in the rhizosphere, as also reported by Zhao et al. (2022). These authors found that silicon-based fertilizers significantly altered soil properties, including reductions in electrical conductivity and pH, even in saline environments. The findings of this study suggest that silicon and potassium act synergistically in alleviating saline stress, creating a more balanced rhizospheric environment. According to previous studies, silicon may modulate the edaphic medium through mechanisms such as complexation of toxic ions, salt adsorption onto colloidal surfaces, or gradual nutrient release, which are widely recognized as a contributing to salt stress tolerance (Coskun et al., 2016; Dhiman et al., 2021; Almeida et al., 2024). In this context, several authors also emphasize the pivotal role of potassium in osmotic regulation and the maintenance of ionic homeostasis under saline conditions (Etesami and Jeong, 2018; El-Egami et al., 2024), making it a valuable strategy for managing salinity.

Moreover, a significant increase in the availability of macro- and micronutrients in the substrate was observed, especially at intermediate to high silicon doses (**Figures 3**, **4**). As highlighted by Greger et al. (2018), the effect of silicon on nutrient availability varies depending on the element, precluding generalizations for all soil ions. According to Matichenkov and Bocharnikova (2001), the increased nutrient availability reported in previous studies following silicon application may be related to the presence of monosilicic acids and the formation of secondary minerals, such as amorphous silica, montmorillonite, and vermiculite, among other factors contributing to the regulation of soil solution chemistry. These mechanisms, although plausible, were not directly measured in the present study.

The enhanced nutrient availability in the soil solution (**Figures 3**, **4**) directly influenced leaf mineral composition, with marked increases in nitrogen, phosphorus, and potassium, as well as efficient accumulation of essential micronutrients in plants under high salinity (**Figures 6**-**9**). The underlying mechanisms for these effects remain hypothetical. According to Khan et al. (2016), silicon’s regulatory action on specific transporters, such as *LSi1*, *LSi2*, and *LSi6*, may enhance nutrient uptake and translocation under stress. Additionally, Khan et al. (2016) suggested that the rise in monosilicic acids in the soil solution and the formation of secondary minerals could enhance cation exchange capacity and maintain nutrients like N and P in more bioavailable forms (Khan et al., 2016). In our study, potassium application also significantly improved mineral nutrition and stress tolerance (**Figures 6**-**9**). Furthermore, the reduction in sodium content in the leaves (**Figure 10A**), concurrent with its decrease in the soil (**Figure 5**), demonstrates that both silicon and potassium play active roles in Na^+^ exclusion and/or compartmentalization, key mechanisms of salinity tolerance in plants, according to Ebeed et al. (2024). This effect was corroborated by the increased silicon content in the leaves (**Figure 10B**), indicating that the element was effectively absorbed and contributed to mitigating physiological damage.

The beneficial effects of Si and K on plant metabolism were also reflected in biochemical and physiological variables (**Figures 11**-**15**). From a biochemical perspective, the significant increase in total chlorophyll, especially at higher Si and K doses (**Figure 11**), indicates improvements that, according Taiz et al. (2017), are typically associated with thylakoid structure and functionality of the photosynthetic apparatus, which is often compromised under high salinity. On the other hand, the decrease in proline content compared to saline controls (**Figure 12**) suggests a reduced need for osmotic adjustment via compatible amino acids, indicating effective stress mitigation, as proline typically accumulates under adverse conditions as a cellular protective mechanism.

The observed increases in stomatal conductance (**Figures 13A, B**), net CO_2_ assimilation (**Figures 13C, D**), and transpiration (**Figure 14C**), especially at the highest treatment doses, demonstrate that both elements enhanced stomatal function and carbon fixation even under saline conditions. The reduction in *C_i_* (**Figures 14A, B**) and *E*, coupled with increased *A*, indicates greater photosynthetic efficiency and improved water-use economy. This functional enhancement aligns with numerous studies in various plant species, which highlight silicon’s role in reducing chloroplast damage under abiotic stress conditions (Qian et al., 2006; Muneer et al., 2014; Cao et al., 2015; Vaculík et al., 2015). Improvements in other physiological traits, such as increased relative water content (**Figures 15A, B**) and reduced electrolyte leakage (**Figures 15C, D**), reflect greater membrane stability and water balance maintenance in plants treated with silicon and potassium.

These physiological enhancements were also reflected in plant morphological parameters. The increases in plant height (**Figures 16A, B**) and in shoot (**Figures 16C, D**) and root (**Figures 16E, F**) biomass accumulation were consistent with improvements in soil chemical properties, mineral nutrition, physiological responses, and osmotic balance. Moreover, the pronounced gains in these variables with increasing Si and K doses confirm the structural and functional roles of these nutrients in promoting plant growth under saline stress. The greatest shoot and root dry mass accumulations were observed at the highest silicon doses, particularly when combined with potassium, confirming the role of both nutrients in mitigating the deleterious effects of salinity. Overall, these results demonstrate that the combined application of Si and K not only alleviates salt-induced physiological constraints but also translates into tangible gains in plant growth and biomass production, supporting similar findings reported by Almeida et al. (2024).

## 5 Conclusion

In summary, our results demonstrate that the combined application of silicic acid (5.04 g dm^-3^) and potassium sulfate (600 mg dm^-3^) is an effective strategy to mitigate salt stress in yellow passion fruit seedlings. Overall, the interrelationships among the variables showed that improvements in soil chemical properties, promoted by silicon and potassium application, led to enhanced physiological and nutritional conditions, favoring the biochemical performance and growth of seedlings irrigated with saline water. Compared to control 2 (non-saline water), although salinity effects were not completely eliminated, treatments with silicon and potassium significantly reduced its adverse impacts. The combined use of Si and K enables the production of vigorous seedlings even under saline irrigation, offering a viable alternative for fruit cultivation in regions increasingly affected by water salinization.

## Data Availability

The raw data supporting the conclusions of this article will be made available by the authors, without undue reservation.
