# Supplementary material for: Silicon and potassium synergistically alleviate salt stress and enhance soil fertility, nutrition, and physiology of passion fruit seedlings
Source: Front Plant Sci. 2025 Oct 21;16:1685221. doi: 10.3389/fpls.2025.1685221 (PMC12583069; doi:10.3389/fpls.2025.1685221)
Supplement: Supplementary file 1 [file DataSheet1.pdf]

STATISTICAL INFORMATIONS

Table 1. Summary of the analysis of variance (mean square) for pH, electrical conductivity (EC), and element contents (phosphorus [P], potassium [K], calcium [Ca], magnesium [Mg], sulfur [S], copper [Cu], iron [Fe], manganese [Mn], zinc [Zn]), and sodium [Na]) in the soil of passion fruit seedling cultivation under varying silicon and potassium doses.

Source of Variation	DF	Mean square											
		pH	EC	P	K	Ca	Mg	S	Cu	Fe	Mn	Zn	Na
Silicon (Si)	3	12.71**	0.86**	48.27**	0.32**	26.54**	2.09**	16,843.06**	0.03**	4,566.95**	2,375.53**	5.28**	4.28**
Potassium (K)	1	3.12*	0.08**	9.56**	0.36**	3.61**	0.22**	16,608.25**	0.004**	3,513.22**	1.05**	8.001**	0.54**
Si × K interaction	3	2.10*	0.03**	1.91^{ns}	0.05**	0.22**	0.36**	2,795.69**	0.008**	1,806.42**	825.01**	21.89**	0.13 ^{ns}
Controls	1	2.74*	7.09**	53.27**	0.09**	1.06**	1.09**	2,228.77**	0.014**	4,146.92**	362.64**	5.84**	43.76**
Controls × treatments	1	84.86**	0.01**	280.94**	3.58**	92.33**	0.159**	177,575.07**	0.0039**	1,891.69**	2,126.15**	0.4**	3.31**
Error	40	0.55	0.002	0.93	0.004	0.04	1.26	275.99	0.0002	55.92	59.84	0.17	0.35
CV		12.40	3.28	3.11	3.29	3.22	4.46	6.42	2.91	9.55	6.41	2.79	16.48

^{ns}: not significant; *: significant at $p < 0.05$; **: significant at $p < 0.01$. according to the F-test; DF: degrees of freedom; CV: coefficient of variation.

Table 2. p-values of F-test for all sources of variation for the variables pH, electrical conductivity (EC), and element contents (phosphorus [P], potassium [K], calcium [Ca], magnesium [Mg], sulfur [S], copper [Cu], iron [Fe], manganese [Mn], zinc [Zn]), and sodium [Na]) in the soil of passion fruit seedlings.

Source of Variation	p-values											
	pH	EC	P	K	Ca	Mg	S	Cu	Fe	Mn	Zn	Na
Silicon (Si)	<.00001	<.00001	<.00001	<.00001	<.00001	<.00001	<.00001	<.00001	<.00001	<.00001	<.00001	0.00001
Potassium (K)	0.08516	<.00001	0.00270	<.00001	<.00001	0.01961	<.00001	0.00001	<.00001	0.89526	<.00001	0.22430
Si × K interaction	0.15532	<.00001	0.12250	0.00003	0.00286	0.00007	<.00001	<.00001	<.00001	<.00001	<.00001	0.77361
Controls	<.00001	<.00001	<.00001	0.00008	0.00001	<.00001	0.11492	<.00001	<.00001	0.01890	<.00001	<.00001
Controls × treatments	0.00332	0.11125	<.00001	<.00001	<.00001	0.00028	<.00001	0.00002	<.00001	<.00001	0.13476	0.00400

Table 3. p-values of Dunnet's test for the variables pH, electrical conductivity (EC), and element contents (phosphorus [P], potassium [K], calcium [Ca], magnesium [Mg], sulfur [S], copper [Cu], iron [Fe], manganese [Mn], zinc [Zn]), and sodium [Na]) in the soil of passion fruit seedlings.

Contrasts	p-values of Dunnett's test											
	pH	EC	P	K	Ca	Mg	S	Cu	Fe	Mn	Zn	Na
Control 1 × Si_1.26_K150	<.0001	<.0001	<.0001	<.0001	<.0001	0.5569	<.0001	0.8348	0.5306	<.0001	1.0000	0.0017
Control 1 × Si_1.26_K600	<.0001	<.0001	<.0001	<.0001	<.0001	0.2307	<.0001	0.8348	0.0024	<.0001	0.7744	<.0001
Control 1 × Si_2.52_K150	<.0001	<.0001	<.0001	<.0001	<.0001	0.0230	<.0001	0.5958	1.0000	0.0002	0.8999	<.0001
Control 1 × Si_2.52_K600	<.0001	<.0001	<.0001	<.0001	<.0001	0.0310	<.0001	0.8348	0.8546	0.0027	0.6908	<.0001
Control 1 × Si_3.78_K150	<.0001	<.0001	<.0001	<.0001	<.0001	<.0001	<.0001	0.0003	<.0001	0.1762	1.0000	<.0001
Control 1 × Si_3.78_K600	<.0001	<.0001	<.0001	<.0001	<.0001	<.0001	<.0001	0.8348	0.6303	1.0000	0.0023	<.0001
Control 1 × Si_5.04_K150	<.0001	<.0001	<.0001	<.0001	<.0001	0.0038	<.0001	0.0003	0.0126	1.0000	<.0001	<.0001
Control 1 × Si_5.04_K600	<.0001	<.0001	<.0001	<.0001	<.0001	1.0000	<.0001	<.0001	0.0002	0.0038	0.1202	<.0001
Control 2 × Si_1.26_K150	<.0001	<.0001	0.6436	<.0001	0.0289	0.2307	<.0001	<.0001	<.0001	<.0001	0.2563	<.0001
Control 2 × Si_1.26_K600	<.0001	<.0001	0.7056	<.0001	<.0001	0.5569	<.0001	<.0001	<.0001	0.0024	0.0009	0.0091
Control 2 × Si_2.52_K150	<.0001	<.0001	0.0254	<.0001	<.0001	0.9846	<.0001	<.0001	<.0001	0.0812	0.8390	0.0006
Control 2 × Si_2.52_K600	<.0001	<.0001	0.0007	<.0001	<.0001	0.9700	<.0001	<.0001	<.0001	0.3875	0.9688	0.0179
Control 2 × Si_3.78_K150	<.0001	<.0001	<.0001	<.0001	<.0001	0.0005	<.0001	1.0000	0.9999	0.9992	0.2563	0.1882
Control 2 × Si_3.78_K600	<.0001	<.0001	<.0001	<.0001	<.0001	0.0074	<.0001	<.0001	<.0001	0.4405	0.9190	0.8826
Control 2 × Si_5.04_K150	<.0001	<.0001	<.0001	<.0001	<.0001	1.0000	<.0001	1.0000	0.1671	0.4093	0.0090	0.3443
Control 2 × Si_5.04_K600	<.0001	<.0001	<.0001	<.0001	<.0001	0.0009	<.0001	0.6706	0.9929	0.3839	<.0001	0.9418

Si_1.26, Si_2.52, Si_3.78, and Si_5.04: doses of 1.26, 2.52, 3.78, and 5.04 g of Si dm^{-3} of soil; K150 and K600: doses of 150 and 600 mg of K dm^{-3} of soil, respectively; Control 1 and Control 2: plants irrigated with water at electrical conductivities of 4.0 and 0.5 dS m^{-1}, respectively.

Table 4. Summary of the analysis of variance (mean square) for nitrogen (N), phosphorus (P), potassium (K), calcium (Ca), magnesium (Mg), sulfur (S), sodium (Na), copper (Cu), iron (Fe), manganese (Mn), zinc (Zn), and silicon (Si) in the leaves of passion fruit seedling cultivation under varying silicon and potassium doses.

Source of Variation	DF	Mean square											
		N	P	K	Ca	Mg	S	Na	Cu	Fe	Mn	Zn	Si
Silicon (Si)	3	457.62**	12.791**	257.63**	15.01**	0.296**	2.61**	86.82**	7.72**	2,142.9**	4,084.68**	44.4**	2,838,607.56**
Potassium (K)	1	456.91**	10.527**	1,703.03**	21.61**	0.99**	1.6**	100.17**	20.02**	419.26**	2,608.23**	147.07**	21.904**
Si × K interaction	3	3.71**	0.772**	127.32**	2.49**	0.05**	0.05ns	1.97**	0.9**	11.89ns	327.16**	17.89**	124,429.88**
Controls	1	3,254.78**	19.937**	390.63**	12.1**	0.4**	0.63**	790.32**	2.03**	474.72**	232.33**	15.63**	14,882.62**
Controls × treatments	1	2,138.38**	4.193**	944.39**	34.78**	0.76**	7.22**	117.19**	44.09**	316.76**	2,100.82**	308.76**	3,413,649.33**
Error	40	0.22	0.024	2.62	0.041	0.009	0.21	1.36	0.003	36.91	7.015	1.365	7,092.99
CV		0.71	5.446	3.98	2.86	2.37	12.58	5.03	0.69	2.79	3.84	2.95	5.004

ns: not significant; *: significant at $p < 0.05$; **: significant at $p < 0.01$. according to the F-test; DF: degrees of freedom; CV: coefficient of variation.

Table 5. p-values of F-test for all sources of variation for the variables nitrogen (N), phosphorus (P), potassium (K), calcium (Ca), magnesium (Mg), sulfur (S), sodium (Na), copper (Cu), iron (Fe), manganese (Mn), zinc (Zn), and silicon (Si) in the leaves of passion fruit seedlings.

Source of Variation	p-values											
	N	P	K	Ca	Mg	S	Na	Cu	Fe	Mn	Zn	Si
Silicon (Si)	<.00001	<.00001	<.00001	<.00001	<.00001	0.00001	<.00001	<.00001	<.00001	<.00001	<.00001	<.00001
Potassium (K)	<.00001	<.00001	<.00001	<.00001	<.00001	0.00883	<.00001	<.00001	0.00180	<.00001	<.00001	0.95599
Si × K interaction	<.00001	<.00001	<.00001	<.00001	0.00587	0.88156	0.24539	<.00001	0.80930	<.00001	0.00001	<.00001
Controls	<.00001	<.00001	<.00001	<.00001	<.00001	0.09208	<.00001	<.00001	0.00099	<.00001	0.00174	0.15613
Controls × treatments	<.00001	<.00001	<.00001	<.00001	<.00001	<.00001	<.00001	<.00001	0.00586	<.00001	<.00001	<.00001

Table 6. p-values of Dunnet's test for the variables nitrogen (N), phosphorus (P), potassium (K), calcium (Ca), magnesium (Mg), sulfur (S), sodium (Na), copper (Cu), iron (Fe), manganese (Mn), zinc (Zn), and silicon (Si) in the leaves of passion fruit seedlings.

Contrasts	p-values of Dunnett's test											
	N	P	K	Ca	Mg	S	Na	Cu	Fe	Mn	Zn	Si
Control 1 × Si_1.26_K150	<.0001	<.0001	<.0001	<.0001	<.0001	0.2793	<.0001	<.0001	0.9975	0.0122	0.9396	0.0005
Control 1 × Si_1.26_K600	<.0001	0.0640	<.0001	<.0001	0.9877	0.8643	<.0001	<.0001	1.0000	0.9531	0.0026	0.7668
Control 1 × Si_2.52_K150	<.0001	0.0150	<.0001	<.0001	<.0001	0.0113	<.0001	<.0001	1.0000	<.0001	0.0652	<.0001
Control 1 × Si_2.52_K600	<.0001	<.0001	<.0001	<.0001	0.5549	0.3109	<.0001	<.0001	0.6454	<.0001	0.0001	<.0001
Control 1 × Si_3.78_K150	<.0001	0.9719	<.0001	<.0001	<.0001	<.0001	<.0001	<.0001	0.1451	0.0003	0.0004	<.0001
Control 1 × Si_3.78_K600	<.0001	<.0001	<.0001	<.0001	0.0002	0.0005	<.0001	<.0001	0.0149	<.0001	<.0001	<.0001
Control 1 × Si_5.04_K150	<.0001	<.0001	<.0001	<.0001	<.0001	0.0047	<.0001	<.0001	0.0002	0.0149	0.2420	<.0001
Control 1 × Si_5.04_K600	<.0001	<.0001	<.0001	<.0001	<.0001	0.0423	<.0001	<.0001	<.0001	<.0001	<.0001	<.0001
Control 2 × Si_1.26_K150	0.0003	<.0001	0.0029	1.0000	0.8102	0.9911	<.0001	<.0001	0.0402	<.0001	0.9997	0.0213
Control 2 × Si_1.26_K600	<.0001	<.0001	0.8183	0.1592	0.0855	1.0000	<.0001	<.0001	0.2915	0.0008	<.0001	1.0000
Control 2 × Si_2.52_K150	0.0239	<.0001	0.0067	<.0001	0.5549	0.3800	<.0001	<.0001	0.3291	<.0001	0.0002	0.0001
Control 2 × Si_2.52_K600	0.9830	<.0001	<.0001	0.0018	0.5549	0.9944	0.0001	<.0001	0.9996	<.0001	<.0001	0.0008
Control 2 × Si_3.78_K150	0.0600	<.0001	1.0000	<.0001	0.0855	0.0003	<.0001	<.0001	1.0000	0.8478	<.0001	<.0001
Control 2 × Si_3.78_K600	0.9830	<.0001	<.0001	0.0003	0.9877	0.0423	0.0145	<.0001	0.9666	<.0001	<.0001	<.0001
Control 2 × Si_5.04_K150	0.2792	<.0001	0.9026	<.0001	0.0055	0.2226	0.0050	0.4765	0.2077	1.0000	0.0016	<.0001
Control 2 × Si_5.04_K600	0.9991	0.8977	<.0001	<.0001	0.5549	0.6968	1.0000	<.0001	0.0099	<.0001	<.0001	<.0001

Si_1.26, Si_2.52, Si_3.78, and Si_5.04: doses of 1.26, 2.52, 3.78, and 5.04 g of Si dm^{-3} of soil; K150 and K600: doses of 150 and 600 mg of K dm^{-3} of soil, respectively. Control 1 and Control 2: plants irrigated with water at electrical conductivities of 4.0 and 0.5 dS m^{-1}, respectively.

Table 7. Summary of the analysis of variance (mean square) for total chlorophyll, proline, stomatal conductance (g_s), net CO_2 assimilation rate (A), intercellular CO_2 concentration (C_i), transpiration rate (E), relative water content (RWC), electrolyte leakage (EL), shoot dry mass (SDM), and root dry mass (RDM) in passion fruit seedling cultivation under varying silicon and potassium doses.

Source of Variation	DF	Mean square										
		Total chlorophyll	Proline	g_s	A	C_i	E	RWC	EL	Plant height	SDM	RDM
Silicon (Si)	3	288.20^{**}	0.77^{**}	0.006^{**}	44.57^{**}	$5,152.41^{**}$	4.34^{**}	397.49^{**}	202.01^{*}	448.32^{**}	2.03^{**}	0.18^{**}
Potassium (K)	1	157.77^{*}	0.54^{**}	0.009^{**}	41.47^{**}	$2,506.99^{**}$	0.54ns	846.49^{**}	545.39^{**}	878.43^{**}	6.59^{**}	0.24^{**}
Si × K interaction	3	3.97^{ns}	0.076^{**}	0.0001^{ns}	4.53^{ns}	384.68^{ns}	1.86^{**}	50.77^{ns}	31.19^{ns}	144.42^{ns}	0.46^{ns}	0.01^{ns}
Controls	1	$1,780.36^{**}$	0.31^{**}	0.012^{**}	192.98^{**}	$1,456.13^{ns}$	10.46^{**}	656.91^{**}	333.97^{*}	$6,200.10^{**}$	76.21^{**}	2.43^{**}
Controls × treatments	1	9.55^{ns}	2.26^{**}	0.002^{ns}	29.87^{**}	$3,775.72^{**}$	1.59^{*}	65.52^{ns}	384.67^{*}	28.16^{ns}	4.17^{*}	0.17^{*}
Error	40	6.85	0.007	0.0008	4.07	466.12	0.25	46.52	73.52	86.74	0.40	0.04
CV		9.01	4.02	22.36	14.95	13.73	14.48	10.10	26.88	17.91	17.11	24.50

^{ns}: not significant; *: significant at $p < 0.05$; **: significant at $p < 0.01$. according to the F-test; DF: degrees of freedom; CV: coefficient of variation.

Table 8. p-values of F-test for all sources of variation for the variables total chlorophyll, proline, stomatal conductance (g_s), net CO_2 assimilation rate (*A*), intercellular CO_2 concentration (C_i), transpiration rate (*E*), relative water content (RWC), electrolyte leakage (EL), shoot dry mass (SDM), and root dry mass (RDM) in passion fruit seedlings.

Source of Variation	p-values										
	Total chlorophyll	Proline	g_s	*A*	C_i	*E*	RWC	EL	Plant height	SDM	RDM
Silicon (Si)	<.00001	<.00001	0.00067	0.00005	0.00020	<.00001	0.00017	0.04455	0.00410	0.00471	0.00536
Potassium (K)	0.00002	<.00001	0.00138	0.00276	0.02558	0.15286	0.00012	0.00894	0.00282	0.00023	0.01380
Si × K interaction	0.63210	<.00001	0.90839	0.35503	0.48774	0.00005	0.36391	0.73665	0.18985	0.33720	0.82098
Controls	<.00001	<.00001	0.00037	<.00001	0.08478	<.00001	0.00055	0.03925	<.00001	<.00001	<.00001
Controls × treatments	0.24478	<.00001	0.08300	0.00992	0.00695	0.01651	0.24236	0.02753	0.57199	0.04765	0.03739

Table 9. p-values of Dunnet's test for the variables total chlorophyll, proline, stomatal conductance (g_s), net CO_2 assimilation rate (A), intercellular CO_2 concentration (C_i), transpiration rate (E), relative water content (RWC), electrolyte leakage (EL), shoot dry mass (SDM), and root dry mass (RDM) in passion fruit seedlings.

Contrasts	p-values of Dunnett's test										
	Total chlorophyll	Proline	g_s	A	C_i	E	RWC	EL	Plant height	SDM	RDM
Control 1 × Si_1.26_K150	0.9277	<.0001	0.9723	1.0000	0.7544	<.0001	1.0000	1.0000	0.1007	0.0697	0.9868
Control 1 × Si_1.26_K600	0.0006	<.0001	0.0399	0.9901	0.9682	0.5631	0.7277	0.3258	0.0431	0.0014	0.2188
Control 1 × Si_2.52_K150	<.0001	<.0001	0.9824	0.9991	0.5616	0.2166	1.0000	0.7798	0.0334	0.0005	0.6269
Control 1 × Si_2.52_K600	<.0001	<.0001	0.3845	0.9954	1.0000	0.4733	0.0270	0.1514	0.0001	<.0001	0.2866
Control 1 × Si_3.78_K150	<.0001	0.2548	1.0000	0.2107	0.1844	1.0000	0.9908	0.5519	0.1583	0.0002	0.4050
Control 1 × Si_3.78_K600	<.0001	0.9852	0.9488	0.0008	0.2810	0.4494	0.0113	0.1078	<.0001	<.0001	0.0355
Control 1 × Si_5.04_K150	<.0001	<.0001	0.9177	0.8658	0.0001	0.9999	0.0083	0.2127	0.0001	0.0023	0.0355
Control 1 × Si_5.04_K600	<.0001	0.8024	1.0000	0.0058	0.0125	0.9991	0.0003	0.0168	<.0001	<.0001	0.0002
Control 2 × Si_1.26_K150	<.0001	<.0001	0.2061	<.0001	1.0000	1.0000	0.0515	0.6011	0.0001	0.0001	<.0001
Control 2 × Si_1.26_K600	<.0001	<.0001	1.0000	<.0001	0.9999	0.0033	0.6481	1.0000	0.0004	0.0070	0.0001
Control 2 × Si_2.52_K150	<.0001	<.0001	0.1765	<.0001	1.0000	0.0185	0.0570	1.0000	0.0005	0.0187	<.0001
Control 2 × Si_2.52_K600	<.0001	<.0001	0.8720	<.0001	0.7255	0.0049	1.0000	0.9991	0.1007	0.1970	0.0001
Control 2 × Si_3.78_K150	<.0001	<.0001	0.0030	0.0057	0.9906	<.0001	0.1895	1.0000	0.0001	0.0413	<.0001
Control 2 × Si_3.78_K600	<.0001	0.0001	0.2595	0.5764	0.9986	0.0054	1.0000	0.9959	0.3969	0.9655	0.0014
Control 2 × Si_5.04_K150	<.0001	<.0001	0.0002	0.0002	0.0142	<.0001	1.0000	0.9999	0.1510	0.0043	0.0014
Control 2 × Si_5.04_K600	0.0086	<.0001	0.0181	0.2001	0.5360	<.0001	0.9367	0.8231	0.5975	0.9981	0.1633

Si_1.26, Si_2.52, Si_3.78, and Si_5.04: doses of 1.26, 2.52, 3.78, and 5.04 g of Si dm^{-3} of soil; K150 and K600: doses of 150 and 600 mg of K dm^{-3} of soil, respectively.
Control 1 and Control 2: plants irrigated with water at electrical conductivities of 4.0 and 0.5 dS m^{-1}, respectively.
