# Supplementary material for: Silicon and potassium synergistically alleviate salt stress and enhance soil fertility, nutrition, and physiology of passion fruit seedlings
Source: Front Plant Sci. 2025 Oct 21;16:1685221. doi: 10.3389/fpls.2025.1685221 (PMC12583069; doi:10.3389/fpls.2025.1685221)
Supplement: Supplementary file 2 [file DataSheet2.pdf]

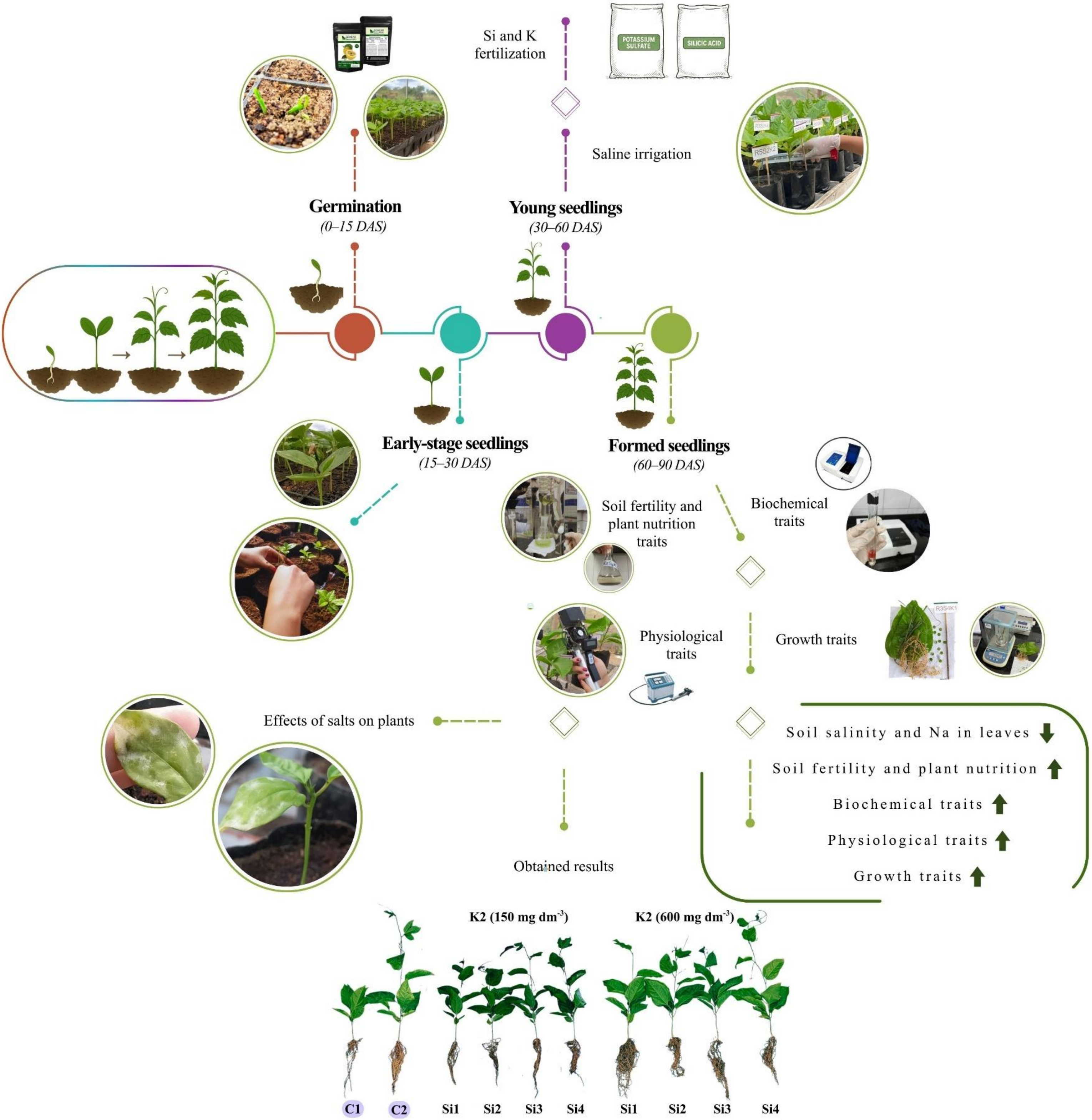

Supplementary Figure. Experimental setup diagram and main phenotypic results. The diagram illustrates the experimental workflow and the effects of silicon (Si1–Si4) and potassium (K1–K2) on alleviating salt stress compared to controls C1 (saline water, EC 4.0 dS m^{-1}) and C2 (low-salinity water, EC 0.5 dS m^{-1}). At the end, plant images highlight the observed phenotypic differences. DAS: days after sowing; Si1–Si4: 1.26, 2.52, 3.78, 5.04 mg Si per dm^3 substrate; K1–K2: 150 and 600 mg K per dm^3 substrate.
